# Perceptions of residents, medical and nursing students about Interprofessional education: a systematic review of the quantitative and qualitative literature

**DOI:** 10.1186/s12909-017-0909-0

**Published:** 2017-05-03

**Authors:** Cora L.F. Visser, Johannes C.F. Ket, Gerda Croiset, Rashmi A. Kusurkar

**Affiliations:** 1 Research in Education Department, VUmc School of Medical Sciences (In affiliation with LEARN! Research Institute for Learning and Education, VU University, Amsterdam, The Netherlands), P.O. Box 7057, 1007 MB Amsterdam, The Netherlands; 2 VUmc Amstel Academie, P.O. Box 7057, 1007 MB Amsterdam, NL The Netherlands; 3 Medical Library, VUmc School of Medical Sciences (In affiliation with LEARN! Research Institute for Learning and Education, VU University, Amsterdam, The Netherlands), P.O. Box 7057, 1007 MB Amsterdam, The Netherlands; 4 Medical Education, VUmc School of Medical Sciences (In affiliation with LEARN! Research Institute for Learning and Education, VU University, Amsterdam, The Netherlands), P.O. Box 7057, 1007 MB Amsterdam, The Netherlands

**Keywords:** Facilitators, Barriers, Readiness for IPE, Affective component of learning process

## Abstract

**Background:**

To identify facilitators and barriers that residents, medical and nursing students perceive in their Interprofessional Education (IPE) in a clinical setting with other healthcare students.

**Methods:**

A systematic review was carried out to identify the perceptions of medical students, residents and nursing students regarding IPE in a clinical setting. PubMed, CINAHL, ERIC and PsycInfo were searched, using keywords and MeSH terms from each database’s inception published prior to June 2014. Interprofessional education involving nursing and medical students and/or residents in IPE were selected by the first author. Two authors independently assessed studies for inclusion or exclusion and extracted the data.

**Results:**

Sixty-five eligible papers (27 quantitative, 16 qualitative and 22 mixed methods) were identified and synthesized using narrative synthesis. Perceptions and attitudes of residents and students could be categorized into ‘Readiness for IPE’, ‘Barriers to IPE’ and ‘Facilitators of IPE’. Within each category they work at three levels: individual, process/curricular and cultural/organizational. Readiness for IPE at individual level is higher in females, irrespective of prior healthcare experience. At process level readiness for IPE fluctuates during medical school, at cultural level collaboration is jeopardized when groups interact poorly. Examples of IPE-barriers are at individual level feeling intimidated by doctors, at process level lack of formal assessment and at cultural level exclusion of medical students from interaction by nurses. Examples of IPE-facilitators are at individual level affective crises and patient care crises situations that create feelings of urgency, at process level small group learning activities in an authentic context and at cultural level getting acquainted informally.

These results are related to a model for learning and teaching, to illustrate the implications for the design of IPE.

**Conclusions:**

Most of the uncovered barriers are at the cultural level and most of the facilitators are at the process level. Factors at the individual level need more research.

**Electronic supplementary material:**

The online version of this article (doi:10.1186/s12909-017-0909-0) contains supplementary material, which is available to authorized users.

## Background

Interprofessional Education (IPE) has been defined as situations “where two or more professions learn with, from and about each other to improve collaboration and the quality of care“ [1]. IPE is considered important to prepare students for successful Interprofessional Collaboration, which is critical for patient safety and quality of care [2, 3]. Numerous IPE initiatives are launched every year all across the globe, but till now there is no generalizable theory which can explain how, why or when learning through IPE is successful [4, 5].

Over the years some theories have been proposed to understand IPE [6]. The Intergroup Contact Theory (ICT) which concerns the ‘learning with and about’ out-groups, states that learners need to overcome prejudice, stereotyped views and discrimination of the group they do not belong to [7]. The theory of social capital suggests that creating sociable relationships between students of different professions during IPE activities leads to trust in other professional groups during practice [6]. The D’Amour model of interprofessionality proposes how IPE initiatives and interprofessional practice are connected and includes the relationships between sociological factors that are involved in IPE and IPC [3]. Still there is no consensus on which theory prevails.

Along with sociological aspects covered in the abovementioned theories, psychological components of learning, like perceptions and processes for learning, can impact the effect of IPE, but have not been investigated. The affective component of learning (motivation and emotion) has been largely neglected in curricular changes in medical education and attention has been mainly directed towards the cognitive and metacognitive components [8].

Perceptions and attitudes are affective factors that contribute to the behavior of students; more specifically strong attitudes guide behavior and weak attitudes follow behavior [9]. When students are expected to exhibit interprofessional collaboration (IPC) in real or simulated health care situations, their attitudes should be strong enough to guide their behavior. For IPE, this implies that when IPC is the learning goal for our students, we need to ascertain the strength of their attitudes rather than (just) observing their behavior.

We were interested in exploring to what extent IPE interventions investigate and integrate the affective component of learning in their designs, so we reviewed the literature to address the research question “What are the perceptions and attitudes of nursing and medical students and residents towards Interprofessional education?”

## Methods

### Study design

A systematic review of the perceptions of students of IPE was performed with a review protocol based on the Preferred Reporting Items for Systematic Reviews and Meta-Analysis (PRISMA)-statement [10]. Due to the diversity of outcome measures, no meta-analysis or additional analyses of risk for bias were performed.

### Data bases and search strategy

PubMed, Ebsco/ERIC, Ebsco/PsycInfo and Ebsco/CINAHL were searched for articles on students’ perceptions of IPE (by CLFV and JCFK). The following terms were used (including synonyms and closely related words) as index terms or free-text words: ‘motivation’ or ‘self-concept’ or ‘attitude’ and ‘interprofessional relations’ and ‘medical or nursing education’. The full search strategies for PubMed, Ebsco/ERIC, Ebsco/PsycInfo and Ebsco/CINAHL is available upon request. In alignment with the Centre for the Advancement of IPE, this paper uses the term IPE to include all such learning in academic and work-based settings before and after qualification [1].

### Study selection

Our aim was to review empirical studies in English, sampling nursing or medical students or residents in a clinical setting or in clinical care, allowing for quantitative, qualitative as well as mixed method studies, using the following inclusion criteria: (a) an IPE initiative where the attitudes or perceptions of students were measured, (b) residents, medical and nursing students as subjects; (c) journal articles only, and (d) empirical studies. In order to assess the quality of the articles, the research team decided to use four items related to methods, results, analysis, and conclusions [11]: 1 = *there is a clear research question/purpose;* 2 = *the method used is suitable for answering the research question;* 3 = *the methods and results are clearly described*; 4 = *the research question is answered in the results and conclusion /discussion sections*. Having indicated the population in our research, we will use ‘students’ when possible to refer to those who are intended to learn with, from and about each other.

First, one author (CLFV) screened all papers obtained from the search for eligibility on their title or abstract. If there was doubt about the content of the study, the abstract (if available) or full-text article was screened and discussed with the other author (RAK). Excluded were studies where only specific medical or specific nursing education was involved (e.g. concerning pharmacology, dental care, mental health care or midwifery and perioperative nurses) as our interest was in the relations of residents, medical and nursing students in generalizable clinical settings. Studies concerning validation of an instrument or evaluation of ‘stand-alone’ e-learning were also excluded when the focus was on their psychometric characteristics rather than the perceptions of students.

Because we aim to unravel the mechanism behind the readiness for IPE, we included mixed methods, quantitative and qualitative studies. By combining the quantitative research with the results from qualitative research, we intend to gain insight into the attitude changes after IPE interventions, thus enabling educators to enhance their IPE by incorporating facilitators and overcoming barriers and to guide the transition of IPE into IPC among students.

### Synthesis of results

In alignment with our research question the findings were synthesized using a qualitative, narrative synthesis. Through this technique, it is feasible to examine empirical studies that differ in their research questions, samples and methods [12] and use text to characterize the findings in multiple groupings [13]. Narrative synthesis appears a stronger method to derive at implications for future research than meta-analysis [14]. CLFV and RAK read through all the papers individually. After reading 5 papers each, they discussed the findings and the emerging themes. They used these themes to extract data from the rest of the papers, adding themes as necessary. After all themes from all papers were extracted, CLFV and RAK individually explored if they could be grouped together into categories (these were Facilitators, Barriers and Readiness). The categories were further grouped according to the naturally emerging levels (i.e. individual, process and cultural). At every step the results were finalized through discussion and consensus in the whole research team. The findings from each study relevant for this review are given in a table (see Additional file 1).

## Results

**Fig. 1:**
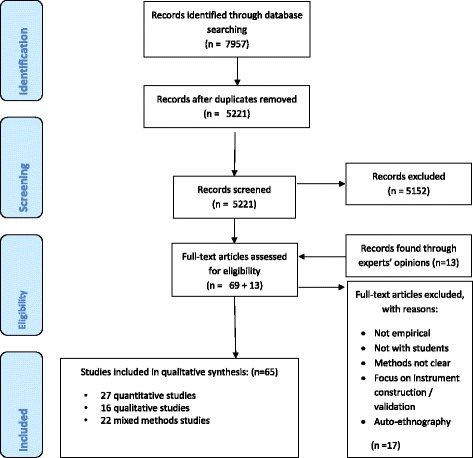
PRISMA 2009 Flow Diagram

The literature search retrieved 7957 articles, of which 65 were included in the review (see PRISMA Flow Diagram - Fig. 1) after removing the duplicates and applying the inclusion and exclusion criteria.

The categories we distinguished in the data were Readiness for IPE, Facilitators of IPE and Barriers to IPE, which were at 3 levels – cultural/ organizational, process/curricular and individual. We will present the results at each of these levels in the abovementioned three categories in this section.

At the cultural or organizational level the factors originate from and are present in the immediate surroundings, are beyond the individual and the curriculum and may be overt or covert. The process or curricular level entails the factors in the curriculum design and delivery. The individual level entails the factors residing within an individual. Readiness for IPE implies the degree of willingness for team-working and team-learning, the significance students give to acquiring their professional identity, the power of their professional culture and the view students hold of professional boundaries or overlap in roles and responsibilities [15]. Facilitators of IPE mean factors that (to some extent) led to achievement of goals and satisfaction of students with the IPE, whereas barriers to IPE mean factors because of which goals were not attained and students were not satisfied. In this section we first report our findings as a combination of a category and a level. We then report a synthesis of the findings across categories and levels.

We present an overview of the number of studies at each level that report readiness for, facilitator of or barrier to IPE in Table 1. Some studies reported both facilitators and barriers, so the total of all studies in Table 1 works out to be more than 65. In the paragraphs following this, the results per category are described at each level in the same sequence as in Tables 2, 3, and 4. The subcategories we distinguish, are in italics.

**Table 1:** Number of studies per level and category (*n* = 65; findings of one study could belong in more than one category)

Level	Cultural	Process	Individual	Total
Category				
Readiness for IPE	1	6	10	17
Facilitators of IPE	4	50	7	61
Barriers to IPE	12	11	4	27
	17	67	21

**Table 2:** Categories and findings - At Cultural / organizational level

Category	Papers	Subcategories	Findings
Readiness for IPE	1 paper [16]	- *Lack of interactions*	- When medical students and nurses do not interact well with the other group, IPC is jeopardized by the associated interpersonal sensitivity score of medical students and hostility score of nurses.
Facilitators	2 papers [17, 19] 1 paper [18]	- *Getting acquainted*	- Time to socialize and experience IPC improved perceptions of IPE. - Students perceived more comfort with approaching non-physicians about patient care issues and understanding of the common challenges non-physicians face.
	1 paper [21]	- *Work experience in health care*	- Positive attitudes towards IPC and perceptions of IPC were maintained and even strengthened once students practiced as qualified professionals.
Barriers	6 papers [16, 22–25, 64]	- *Belonging to social group*	- Medical students perceived nurses to have a less positive status in society, associated some tasks with nurses’ work and refused to do them in the ITU.
	2 papers [17, 19]		- Not knowing students from the other professions meant that time was needed to familiarize.
	1 paper [26]	- *IPC importance stated, not experienced*	- Dissonance between what faculty stated and educational practice
	1 paper [27]	- *Mis-communication*	- Nurses perceived that residents didn’t want to share decision making and vice versa.
	1 paper [28]	- *Work experience in healthcare practice*	- Attitude towards healthcare teams was significantly poorer in students around one year after graduation, in comparison with 3rd year students after the same IPE training.
	1 paper [70]	- *Gaps in role-perception*	- Gaps in perception of the others’ roles was negatively related to attitudes toward collaborative patient care decision making.

**Table 3:** Categories and findings- At Process / curricular level

Category	Papers	Subcategories	Findings
Readiness for IPE	6 papers [29–32, 62, 63]	- *Readiness fluctuates*	- Readiness for IPE and professional identity were highest at entry, higher in students with prior IPE experience and declined significantly over time.
Facilitators	4 papers – workplace setting [35]; practice immersion [34]; 1 day workshop [73]; simulation [33]	- *Immersion in collaboration*	- Diverse IPE forms, all authentic patient settings, improved the perceptions about interpersonal skills, professional competence, leadership, academic ability, being a team player or independent worker, confidence, decision-making and practical skills of the other health professions.
	2 papers [28, 50]		- Understanding of team roles and team interdependence scored high. In one study perception of team efficacy decreased after graduation.
	3 papers [20, 49, 50];	- *Exposure to IP teams*	- Students ask for longitudinal integrated IPE and longitudinal clerkships
			- IPE as a training in comparison with lectures resulted in significantly higher mean scores on the subscales ‘quality of care’ and ‘patient-centered care’.
	7 papers [33, 35, 39–41, 57, 71]	- *Stimulating teamwork training*	- Training of team communication skills enhances motivation and positive attitudes toward IPC. Students had learned about their performance and lack of professional skills alongside team skills.
	6 papers [17, 18, 25, 42–44]	- *Teacher facilitating reflection*	- A teacher helping students think, plan, do and check their work, thus stimulating teamwork rather than teaching knowledge.
	6 papers [23, 24, 46–49, 76]	- *Shared learning*	- Value in learning about professional differences and identity. Students saw the benefits of shared learning, medical students saw the advantages only early in their training.
	18 papers [21, 22, 34, 35, 37, 39, 43, 44, 54–59, 68, 74, 75, 77]	- *Learning in authentic context*	- Clinical realism, like simulation or interprofessional training unit, offered students an opportunity to identify other professionals’ functions in relation to patient care and to clearly assess and describe patients’ problems and needs.
	1 paper [49]		- Readiness for IPE and attitude towards health care teams improved after IPE involving teaching through practice and decreased after IPE involving teaching only through lectures.
	2 papers [43, 77]	- *Integrating IPE & specific learning goals*	- Combination of professional specific and IPE learning goals was achieved by students in advanced years (8th semester)
	1 paper [59]	- *Follow up training*	- A Team communication training was followed by regular IP team meetings.
Barriers	8 papers [17, 22–24, 30, 60, 75, 78]	- *Combining IPE & profess. Specific learning objectives*	- Medical students experienced confusion and tension when profession-specific and IPE objectives are combined.
	1 paper [44]	- *Teacher who just transmits knowledge*	- Teacher who just transmits knowledge rather than stimulating students to think, plan, do and reflect.
	1 paper [23]	- *Lack of assessment*	- IPE loses importance when not assessed, especially for medical students, who are concerned about learning inappropriate skills.
	1 paper [75]	- *Being present in the ward all day*	- Medical students were not used to the requirement to be present in the ward all day.

**Table 4:** Categories and findings- At Individual level

Category	Papers	Subcategories	Findings
Readiness for IPE	4 papers [30, 59, 62, 63]	- *Gender*	- Females had more readiness for IPE than males.
	2 papers [61, 64]	- *Stereotyped views*	- Increased understanding of others’ role and of the students’ own competence in IPC led to lesser stereotyping and more readiness for IPC.
	1 paper [55]		- First year students with a parent working in healthcare started with lower readiness.
	2 papers [30, 63]	- *Earlier healthcare experience*	- Earlier work experience in health care did not influence attitudes toward collaboration but it did result in higher readiness for IPE.
	1 paper [65]	- *Profession and phase of study*	- Younger students achieved more learning outcomes than students who had graduated in some professions.
Facilitators	1 paper [66]	- *Being available and receptive*	- Working together required physical proximity (place), time to interact and intellectual availability, with knowledge about the work they are doing and about each other’s’ roles as care providers. Being receptive is conveying respect, trust and interest in collaboration.
	2 papers [16, 67]	- *Relatedness within/outside group*	- Professionals interact the best in their group, this was overcome when situations created a feeling of urgency and demanded collaboration.
	4 papers [33, 68, 69, 76]	- *Having own role clarity*	- All students groups reported a gain in understanding of the importance of communication and teamwork in patient care; medical students reporting the greatest gain and they also gained most in clarity of own professional role.
Barriers	2 papers [66, 78]	- *Unprofessional behavior*	- Arrogance or disinterest, aggressive behavior, nurses delaying acting on orders or going to other doctors, the need to “sell oneself” to other professions.
	2 papers [40, 53]	- *Emphasizing professional knowledge at the cost of teamwork skills*	- When medical students focus on professional knowledge rather than on teamwork skills, and when nurses feel intimidated.

### Cultural level

#### Readiness for IPE at cultural level

##### Lack of interactions

The low quantity and poor quality of the interactions between medical students and nurses jeopardized their collaboration. Medical students scored high on interpersonal sensitivity (feelings of personal inadequacy and inferiority) compared to nurses. Nurses scored high on hostile thoughts, feelings and actions compared to medical students [16].

#### Facilitators at cultural level

##### Getting acquainted

IPE was facilitated when there was time for different professional groups to meet informally, [17] to get to know members of other professions [18, 19] and by being immersed in an IP-team [20].

##### Work experience in healthcare practice

Positive attitudes towards IPC and perceptions of IPC were maintained and even strengthened once students practiced as qualified professionals [21].

#### Barriers at cultural level

##### Belonging to a social group

Medical students perceived nurses to be more caring than doctors, but as having a less positive status in society and being less competent and academically weaker than doctors [16, 22–25] Not knowing students from the other professions meant that time was needed to familiarize [17, 19].

##### IPC importance stated, but not experienced

The graduates (nursing, medical and pharmacy) reflected and reported that faculty advocated IPE and IPC, but graduates did not actually witness IPC in practice [26].

##### Miscommunication

Expectations of residents and nurses regarding each other’s roles in patient management were similar, namely to be informed. Nurses expect the residents to explain their actions and decisions, to consider nurses’ opinions and recognize nurses’ work. Residents expect nurses to understand the clinical situation, exchange information and participate in establishing a common goal for patient management. Furthermore, residents expect nurses to verify prescriptions and medical decisions. But both groups do not perceive that their expectations are met [27].

##### Experience in healthcare practice

Compared to third year students, the team efficacy was significantly poorer in students one year after graduation, [28] thus negatively influencing their attitude toward collaborative practice.

##### Gaps in role perception

Nursing students had a more accurate perception of the medical role than did medical students of the nursing role. A more accurate role perception was associated with a more positive attitude towards collaborative physician-nurse decision making [25]. Lack of participation in team duties, which medical students described as ‘nursing work’, was a barrier for IPE [17].

Overall at the cultural level, being unfamiliar with the other professions and having stereotypical views of professions stood in the way of IPE. Remarkably, ‘experience in healthcare practice’ appeared to work as a facilitator as well as a barrier.

### Process or curricular level

#### Readiness for IPE at process level

##### Readiness for IPE fluctuates

Readiness for IPE was high at the beginning and decreased from year 1 to the end of the education [29]. A small but significant positive relationship between professional identity and Readiness for IPE was maintained over time [30]. Medical students scored lower on readiness for IPE than nursing students, but higher on the perceived need for collaboration and the actual collaboration [31]. The use of simulation or standardized patients in IPE had a positive effect on readiness for IPE [32].

#### Facilitators at process level

##### Immersion in collaboration

Students perceived that in an Interprofessional Training Unit (ITU) they learned to identify the functions of other professions in relation to inpatient care, to clearly assess and describe patients’ needs and problems, so that other professions can engage in a dialogue about goals and actions [33–37].

##### Exposure to IP teams

After IPE, students of all professions had a more positive perception of the other professions regarding their “subservient” (items: valuing autonomy, assertiveness and technical focus) and “caring” behaviour (items: empathy, approachability and teamwork) [38].

##### Stimulating teamwork training

Training in team communication skills acted as a facilitator by increasing motivation, positive attitude towards IP team communication and perception of utility of IPE [39]. Students expressed that in a team training they had learned about their own performance and their lack of certain professional competencies as well as team skills [40]. Clustering students according to their approaches to learning resulted in a ‘low collaborative group’ of students who, while learning about other professions and their own professional role, did not change their opinion regarding IPE and were not satisfied with it, a ‘collaborative constructivist’ group, where students expect to build their knowledge together with other students and with teachers, and a ‘cookbook’ group. Students with the ‘cookbook style’ had as main conception to acquire definite knowledge from textbooks or from a teacher. The last two groups showed an increased understanding of IPC [41].

##### Teacher facilitating reflection

Students indicated that the role of a facilitator was to make students think, plan and do the work as a part of an Interprofessional Training Unit [42]. Where an interprofessional group had one or more facilitating health care professionals, students perceived them as helpful when they were from their own and other professions [25]. Participation of the preceptor was a facilitator of IPE [18]. A teacher directing questions from a student to another student from the appropriate profession for answering the question, facilitated IPE [43]. A teacher working with the students stimulating teamwork [17] rather than imparting knowledge to them also facilitated IPE [44].

##### Shared learning

In this theme, there were contrasting findings. Several studies found that students see the benefits of shared learning [45, 46], specifically medical students see the advantages at an early stage of their training [24, 30], followed by a decline in readiness for IPE [23, 24, 47]. Students who had undergone Shared Learning followed by an IP clinical placement had better attitudes or more readiness for IPE in comparison with only or no Shared Learning experience [23, 48]. Another study found that an IPE was not effective (because the mean score did not change significantly from that at entry to the course) in spite of a significant increase in the positive attitude to health care after practice training in year 3 as compared to didactic training in year 1 [49].

##### Learning in authentic context

Simulation-based IPE increased self-efficacy and understanding of others’ professional role, [21, 50, 51] indicating that readiness for IPE increased [52, 53]. IPE improved the perceptions of interprofessional teamwork and communication and brought improvement in IP relationships [54–57]. Students who were working in IP groups and were required to complete a root cause analysis and develop recommendations based on an event case, enjoyed working with students from other professions [58]. Interview analysis after an IP simulation training in resuscitation skills showed broad support for IPE matched to clinical reality with perceived benefits for teamwork, communication and improved understanding of roles and perspectives [22].

##### Integration of IPE and profession specific learning goals

Findings from Jakobsen indicate that profession specific knowledge and capability can be learnt alongside interprofessional collaboration by 8th semester medical students [43].

##### Follow up training

A communication training for nurses and residents, followed by weekly meetings of the IP team, showed improvement in attitude toward physician-nurse collaboration and communication [59].

#### Barriers at process level

##### Combination of professions specific and IPE learning objectives

The combination of profession-specific and IPL objectives to be achieved in the same placement created confusion and tension about what to focus on [17, 22–24, 30, 60].

##### Teacher who just transmits knowledge

Students indicated that a teacher just transmitting knowledge was counterproductive for their IPE intervention [44].

##### Lack of assessment

Medical students were reluctant to value learning that is not discipline-specific and particularly if it is not assessed [23]. Concerns of 2nd year medical students were adoption of inappropriate roles, difficulties in scheduling of the intervention and that others would encroach on their professional tasks (e.g. prescribing) [61].

Overall at the process level, readiness for IPE fluctuated during the years of training. Active participation and more self-guided learning of students in the IPE activity led to more satisfaction and improvement of the perceptions of other professions. In one study [43] medical students felt they could achieve the profession specific goals in combination with the IPE goals on the Interprofessional Training Unit (ITU), whereas the findings in 6 papers [17, 22–24, 30, 60] indicate that medical students experienced confusion and stress, but this could well have to do with the philosophy which the Danish ITU held that “emphasis is put on training *professional competency because this is considered necessary for Interprofessional collaboration*”.

### Individual level

#### Readiness for IPE at individual level

##### Gender

Readiness for IPE was higher in female than in male students [30, 62, 63].

##### Stereotyped views

Students entered their health profession education with stereotyped views of other professions, [64] especially when students had a parent working in health care. These views appeared to become more exaggerated during a Common Foundation Programme [61]. Auto-stereotyping is also positively associated with Readiness for IPE [61].

##### Earlier experience in health care

Did result in higher readiness for IPE, [30] but did not influence attitudes toward actual collaboration [63].

##### Phase of study

Younger students (18–24 years) achieved more learning outcomes (e.g. increased knowledge and appreciation of team working) and were more satisfied with the intervention as compared to older students (>25 years) [65].

#### Facilitators at individual level

##### Availability and receptiveness

Nurses and residents indicated that working together is facilitated by physical proximity and conveyance of trust, respect and interest in collaboration [66].

##### Relatedness within and outside own group

Relatedness is the basic psychological need to perceive that one belongs to a group, associated with open interaction. In health care teams, medical students were perceived to interact the best with residents and the worst with nurses, scoring higher on feelings of inferiority and personal inadequacy than nurses. Conversely, nurses interacted the best with other nurses and the worst with medical students, displaying more hostile feelings, thoughts and actions than medical students [16]. In another study, these feelings could be overcome when situations created a feeling of urgency, through patient deterioration or a personal crisis of the individual [67].

##### Having own role clarity

All students groups reported a gain in understanding of the importance of communication and teamwork in patient care, with the medical students gaining most in clarity of own professional role [68, 69].

#### Barriers at individual level

##### Unprofessional behavior

Arrogant and aggressive behavior were barriers to IPE, sometimes resulting in nurses who, when they disagreed with the order, went over the residents’ head to get orders changed. In such cases, nurses felt that they were protecting the patient [66].

##### Emphasizing professional knowledge at the cost of teamwork skills

In an Interprofessional Training Unit medical students tended to focus on professional knowledge and on learning profession specific skills rather than team work skills [40]. Nurses or nursing students felt intimidated by some doctors [53].

Overall at the individual level the requirements for learning their own professional skills and existing stereotyped views of the students, can stand in their way of IP learning.

#### Findings across categories and levels

##### Evaluating learning outcomes

In our review, 14 studies measured the change in attitudes and perceptions [19, 23, 24, 29, 38, 39, 41, 48, 50, 53, 57, 62, 63, 70] pre and post intervention quantitatively with a single instrument. Eight studies applied a combination of two or more questionnaires. [30, 32, 40, 47, 49, 58, 61, 65] A post-intervention interview, focus group or writing assignment offers the student an opportunity to reflect on the interprofessional experience, as was done in 16 studies. [25, 27, 32, 34, 37, 43–45, 51, 53–55, 65, 69, 71, 72] More than 15 different instruments or their adaptations were applied in the 65 studies in this review (see Additional file 1). Twenty-one studies had student-led wards or standardized patients. [17, 27, 32–34, 36–38, 42, 43, 54, 60, 68, 69, 71–77] Only 7 out of 65 studies had assessment incorporated in the design. [22, 25, 38, 57, 58, 74, 78] Especially medical students take learning goals more seriously, when they are assessed [23].

##### Evaluation approach

Five studies applied mixed methods and a longitudinal approach [17, 32, 33, 42, 77].

##### Contact between groups

Several subthemes which could be viewed in the light of the Intergroup Contact Theory were found, but in a very small number of studies: at the *cultural level* readiness for IPE is lower when there is a low frequency of and poor quality in the interactions between professions [16] or when there are gaps in the role perceptions [70]. Getting informally acquainted with each other is a facilitator of IPE [17, 20] and the relatedness within/outside a group was found to be a facilitator in 2 studies [66, 67]. Miscommunication [27], along with emphasizing professional knowledge rather than team work skills [40, 53] are barriers to IPE. First year medical students perceived nurses to have a less positive societal status [24] and post-graduate students highlighted aspects of status differences [25]. In one study medical students felt they had the same status as others students [43]. Three studies explored the stereotyped views that students hold of other professions [35, 61, 64]. In an elaboration of her study [60], Lidskog found that students from occupational therapy and social work see nurses as responsible for holding all parts together, but that sometimes nurses take over, helping the patient more than is needed [79].

##### Adult learning and scaffolding

We could distinguish several subthemes that can be related to scaffolding, problem solving and facilitation of group processes. At the *process level* readiness is highest at the beginning of the education and is enhanced by the development of professional identity and the use of authentic situations for learning [30, 31, 36, 55, 62, 63, 68, 72]. Evidence of facilitating factors for IPE *at process level* is quite strong and could be labelled task-centered orientation: immersion in collaboration is found in 4 papers in different forms [35, 49, 52, 80] and teachers encouraging reflective practice in 4 papers, [17, 18, 25, 42] stimulating teamwork training in 7 papers [21, 22, 35, 39, 40, 43, 56]. Barriers were conflict between profession-specific and interprofessional interests in 6 papers, [17, 22–24, 30, 32]; lack of assessment of IPL [23] and teachers merely transmitting knowledge [44].

## Discussion

In this review, facilitators of and barriers to Interprofessional learning were found on three levels: cultural, process and individual. Most studies in our review have explored interventions at the process level and have mainly described the facilitators, as can be seen in Table 1. At the cultural level, experience in health care practice can work as a facilitator as well as a barrier. Barriers at the process level and at the individual level seem to be underreported. Findings at the individual level reveal higher readiness for IPE in females, in students below 25 years of age, in students who think positively about themselves in their own profession and have prior experience in health care. At the individual level, facilitators of IPE are conveyance of respect, trust and role clarity among professions. Emphasis on professional knowledge rather than team work skills or arrogance are barriers to IPE. These findings together substantiate the role of affective factors in learning for IPC.

This review focused on the affective domain which we believe is a prerequisite for the long-term success of IPE, meaning that it leads to IPC. Although most published IPE initiatives have shown changes in self-reported perceptions or attitudes as outcomes of learning, the mechanisms of the changes have remained largely unexplained, because the evaluation was at a level that is not suited to give an insight into these mechanisms.

### Evaluating learning outcomes

In their analysis of survey instruments used to evaluate IPE initiatives, Gillan et al. found that researchers mainly employed questionnaire items measuring outcomes that could be linked to Kirkpatrick Level 2a [81]. Thistlethwaite and Moran reviewed 40 studies where the IPE learning outcomes are linked to Kirkpatrick Level 2b which indicates ‘Enhanced understanding of roles and responsibilities of other health and social care professionals, improved knowledge of the nature of multidisciplinary teamwork and development of teamwork skills’ [82]. If we want to evaluate whether IPE has actually resulted in IPC (Kirkpatrick’s level 3), questionnaire items should be constructed to assess the learning outcomes in which the individuals transfer their interprofessional learning to their practice [83]. None of the studies in our review claimed to evaluate students at Kirkpatrick level 3 (IPC behaviour), leaving the reader with no answer about the extent to which IPE had led to IPC. The Kirkpatrick level of evaluation is indicated in Additional file 1 (last column).

### Evaluation approach

The five studies that applied mixed methods and a longitudinal approach “come away from an evaluation approach with a focus on outcomes and short term pre and post measurement”, as recommended by Thistlethwaite in an exploratory review [84].

The findings of three studies on stereotyped views are corroborated by Hind [85], who found that early in the course of the study, auto-stereotyping is associated with hetero-stereotyping, meaning that students who think positively about themselves in their own professions also think positively about students in other professions [35, 61, 64]. Although for the Intergroup Contact Theory as well as the adult learning theories several subthemes could be distinguished in our findings, we need a model which is at the intersection of the sociological and psychological aspects of learning to discuss the implications for practice and research.

### Implications for practice and research

Teachers wanting to integrate facilitators of IPE in their intervention may question how to give attention to the affective domain. To bring to light such practical implications, we place our findings in the context of the Learning-Oriented Teaching (LOT) model, which is at the intersection of psychological and sociological aspects of learning and would be an interesting model to relate to the design and implementation of IPE. The LOT model, as suggested by the name, visualizes teaching from the orientation of the learner or student. It combines two aspects of learning: the components of learning and the amount of guidance required [86]. The components of learning are cognition (content of learning), affect (motivation and emotions) and metacognition (Metacognition allows people to take charge of their own learning: awareness of how they learn, evaluation of their learning needs, generating strategies to meet these needs and then implementing the strategies [87]). The amount of guidance given should be customized to the need of each student and may vary from completely by the teacher, via shared with teacher or peers, to completely by the student**.** We place the facilitators at cultural and process level from our review in the LOT model (Table 5 – adapted from. [86]).

**Table 5:** LOT model for Guidance of the learning process in IPE. (adapted from Table 4 of Ten Cate et al. [86])

Source of guidance of the learning process			
Learning process component	Full external guidance (from the teacher only)	Shared guidance (from students and teacher both)	Full internal guidance (from the student only)
Cognitive level			
Learner: what to learn?	Learning with and about others in classroom situation - *Shared learning early in training* - *Role clarity*	Learning from others (roles and responsibilities) - *Getting acquainted*	Learning from other students and patients - *Follow up training* - *Stimulating teamwork training* - *Learning in authentic context*
Teacher: what to present to the student?	Using examples of complex patient problems - *Learning in authentic context (simulation)* Assessment - *Assessment of IPE and specific learning goals*	Facilitating students to think and plan a collaborative approach - *Teacher facilitating reflection* Assessment - *Assessment of IPE and specific learning goals*	Facilitating team work - *Immersion in collaboration* Assessment - *Assessment of integrated IPE and specific learning goals*
Affective level			
Learner: why learn?	Shared learning about patients’ problems - *Inform others about one’s roles & responsibilities*	Learning with others to solve patients’ problems	Reflect on quality of care and patient safety - *Patient problems clear?* - *Team communication*
Teacher: how to motivate the student?	Expose stereotyped views - *Discuss perception of characteristics,* e.g. *professional competence, academic ability*	Give active, patient centred assignments - *Case, simulation* - *Teacher facilitating reflection*	Learning in authentic context - *Integration of specific and IPE learning goals* - *Stimulating teamwork training*
Metacognitive level			
Learner: how to learn?	Learning goals are assessed^a^	Integrating profession specific + IPE goals^b^	Peer coaching
Teacher: how to instruct the student?	- Assessment at cognitive level and reflection for affective level	- *Follow up of teamwork skills* as formative assessment by peers	- (Self-)Assessment with reflection and portfolio

^a^Examples of learning goals assessment in the included papers are: Final class presentation [58]; Judgement by IP facilitators using a rubric at end of placement [74]; Asking for 3 statements about learning in IP Training Unit [43]; Faculty and Standardized Patient using a Teamwork Global Rating Scale [80]

^b^With integrated profession specific and IPE objectives, assessment can be considered a form of guidance as stated by Broadfoot (Broadfoot, Patricia (2007) Introduction to assessment. London: Continuum, p. 135–136): “Self-assessment, therefore, is not really just an assessment practice; it is actually a learning activity. It is a way of encouraging students to reflect on what they have learned so far, to think about ways of improving their learning and to make plans which will enable them to progress as learners and to reach their goals. […] As such it incorporates the skills of time-management, action-planning, negotiation, interpersonal skills, communication - with both teachers and fellow students - and self-discipline in addition to reflection, critical judgment and evaluation”

We incorporated the facilitators found at cultural and process level (indicated with – *italic*, from our Tables  1, 2 and 3 in the column ‘Subcategories’). Since ‘Assessment’ can be considered a form of guidance (Crooks, 1988) and it was missing in most IPE interventions (barrier at process/curricular level), we added it at the cognitive and meta-cognitive level

### Assessment as guidance

Considering the maxim ‘assessment guides the learning’ [88] we add ‘assessment’ to the 3 columns of the LOT model. Under the condition of full internal guidance, assessment would concern self-assessment or reflection. Together with planning and monitoring, these are processes for learning (metacognition). Reflection on the IPE experience through a post-intervention interview, focus group or writing assignment, can in our opinion function as an intervention in itself, reinforcing the value of IPE among the students.

Furthermore, referring to our statement in the Introduction that the strength of the attitudes should be evaluated, we might expect that students who conduct self-guidance of IPE might come closer to IPC, and their learning outcomes can (and in our opinion should) be evaluated at that level, e.g. by employing items that can be linked to Kirkpatrick Level 3.

It is noteworthy that, although we reviewed only perceptions and attitudes for IPE (the affective domain) of students, we are able to make suggestions at the cognitive and metacognitive level. This is because, in our opinion, incorporating elements like assessment and different sources of guidance at the cognitive and metacognitive domains which can appeal to the affective domain, may create more lasting and effective changes at the process level. However, educational researchers in the medical field have not acknowledged the importance of affective factors such as customized guidance on learning and performance [89].

The use of the LOT model can help educators to carefully design IPE with integration of several facilitators of IPE and a conscious choice of the level of evaluation of learning outcomes. Thus, this narrative synthesis has brought us closer to “the ingredients and the mix in the recipe for effective IPE” [90].

This review, and especially Table 1, leads us to the following further research questions in order to connect IPE with IPC, as specified by the D’Amour model of interprofessionality [3]: What are the barriers to IPE at the process level? What are the facilitators and barriers to IPE at the individual level? What are the mechanisms that lead readiness for IPE to IPC behavior? How can readiness for IPE be facilitated?

### Limitations

We chose at the outset to include only papers where medical and nursing students were among the participants and in a clinical setting. We could possibly have found more facilitators and barriers if we had included studies exclusively on other health care students and other settings as well. We excluded an auto-ethnography because we did not find comparable studies [91]. We did so with regret, especially since the affective level was well-represented.

## Conclusions

In this literature review we found that facilitators of IPE at the process level are reported the most and barriers are relatively underreported. Investigating the facilitators and barriers at the individual level and facilitators at cultural level needs attention. We propose that integration of the affective domain in planning, delivering and assessing IPE could be a useful learning oriented approach of teaching interprofessional skills and attitudes.

## Additional file

Additional file 1: Table of papers included in the review. For each included paper the study reference, country, setting, number of students, research objectives, findings relevant to the review, type of study and pedagogical approach are provided. (DOC 226 kb)

## References

[1] Principles of interprofessional education [https://www.caipe.org/download/barr-low-2011-principles-interprofessional-education]

[2] World Health Organization Department of Human Resources for Health (2010). Framework for action on interprofessional education and collaborative practice.

[3] D'Amour D, Oandasan I (2005). Interprofessionality as the field of interprofessional practice and interprofessional education: an emerging concept. J Interprof Care.

[4] Hammick M, Freeth D, Koppel I, Reeves S, Barr H (2007). A best evidence systematic review of interprofessional education: BEME guide no. 9. Med Teach.

[5] Reeves S, Goldman J, Gilbert J, Tepper J, Silver I, Suter E, Zwarenstein M (2011). A scoping review to improve conceptual clarity of interprofessional interventions. J Interprof Care.

[6] Hean S, Craddock DF, Hammick MF, Hammick M (2012). Theoretical insights into interprofessional education: AMEE guide no. 62. Med Teach.

[7] Pettigrew TF (1998). Intergroup contact theory. Annu Rev Psychol.

[8] Kusurkar RA, Croiset G, Mann KV, Custers E, ten Cate O (2012). Have motivation theories guided the development and reform of medical education curricula? A review of the literature. Acad Med.

[9] Holland RW, Verplanken B, Van Knippenberg A (2002). On the nature of attitude-behavior relations: the strong guide, the weak follow. Eur J Soc Psychol.

[10] Preferred Reporting Items for Systematic Reviews and Meta-Analyses (PRISMA). 2009. [http://prisma-statement.org]

[11] Buckley S, Coleman J, Davison I, Khan KS, Zamora J, Malick S, Morley D, Pollard D, Ashcroft T, Popovic C (2009). The educational effects of portfolios on undergraduate student learning: a best evidence medical education (BEME) systematic review. BEME guide no. 11. Med Teach.

[12] Green BN, Johnson CD, Adams A (2006). Writing narrative literature reviews for peer-reviewed journals: secrets of the trade. J Chiropr Med.

[13] Booth A, Sutton A, Papaioannou D (2016). Systematic approaches to a successful literature review.

[14] Rodgers M, Sowden A, Petticrew M, Arai L, Roberts H, Britten N, Popay J (2009). Testing methodological guidance on the conduct of narrative synthesis in systematic reviews effectiveness of interventions to promote smoke alarm ownership and function. Evaluation.

[15] Parsell G, Bligh J (1999). The development of a questionnaire to assess the readiness of health care students for interprofessional learning (RIPLS). Med Educ.

[16] Nadolski GJ, Bell MA, Brewer BB, Frankel RM, Cushing HE, Brokaw JJ (2006). Evaluating the quality of interaction between medical students and nurses in a large teaching hospital. BMC Med Educ.

[17] Reeves S, Freeth D, McCrorie P, Perry D (2002). 'It teaches you what to expect in future ... ': interprofessional learning on a training ward for medical, nursing, occupational therapy and physiotherapy students. Med Educ.

[18] Pathak S, Holzmueller CG, Haller KB, Pronovost PJ (2010). A mile in their shoes: interdisciplinary education at the Johns Hopkins University School of Medicine. Am J Med Qual.

[19] Mitchell M, Groves M, Mitchell C, Batkin J (2010). Innovation in learning - an inter-professional approach to improving communication. Nurse Educ Pract.

[20] Myhre D, Woloschuk W, Pedersen J (2014). Exposure and attitudes toward interprofessional teams: a three-year prospective study of longitudinal integrated clerkship versus rotation-based clerkship students. J Interprof Care.

[21] Pollard KC, Miers ME (2008). From students to professionals: results of a longitudinal study of attitudes to pre-qualifying collaborative learning and working in health and social care in the United Kingdom. J Interprof Care.

[22] Bradley P, Cooper S, Duncan F (2009). A mixed-methods study of interprofessional learning of resuscitation skills. Med Educ.

[23] Morison S, Jenkins J (2007). Sustained effects of interprofessional shared learning on student attitudes to communication and team working depend on shared learning opportunities on clinical placement as well as in the classroom. Med Teach.

[24] Rudland JR, Mires GJ (2005). Characteristics of doctors and nurses as perceived by students entering medical school: implications for shared teaching. Med Educ.

[25] Stepney P, Callwood I, Ning F, Downing K (2011). Learning to collaborate: a study of nursing students' experience of inter-professional education at one UK university. Educ Stud.

[26] Gilligan C, Outram S, Levett-Jones T (2014). Recommendations from recent graduates in medicine, nursing and pharmacy on improving interprofessional education in university programs: a qualitative study. BMC Med Educ.

[27] Muller-Juge V, Cullati S, Blondon KS, Hudelson P, Maitre F, Vu NV, Savoldelli GL, Nendaz MR (2013). Interprofessional collaboration on an internal medicine ward: role perceptions and expectations among nurses and residents. PLoS One.

[28] Makino T, Shinozaki H, Hayashi K, Lee B, Matsui H, Kururi N, Kazama H, Ogawara H, Tozato F, Iwasaki K (2013). Attitudes toward interprofessional healthcare teams: a comparison between undergraduate students and alumni. J Interprof Care.

[29] McFadyen AK, Webster VS, Maclaren WM, O'neill MA (2010). Interprofessional attitudes and perceptions: results from a longitudinal controlled trial of pre-registration health and social care students in Scotland. J Interprof Care.

[30] Coster S, Norman I, Murrells T, Kitchen S, Meerabeau E, Sooboodoo E, d'Avray L (2008). Interprofessional attitudes amongst undergraduate students in the health professions: a longitudinal questionnaire survey. Int J Nurs Stud.

[31] Keshtkaran Z, Sharif F, Rambod M (2014). Students' readiness for and perception of inter-professional learning: a cross-sectional study. Nurse Educ Today.

[32] Solomon P (2011). Student perspectives on patient educators as facilitators of interprofessional education. Med Teach.

[33] Curran VR, Mugford JG, Law RM, MacDonald S (2005). Influence of an interprofessional HIV/AIDS education program on role perception, attitudes and teamwork skills of undergraduate health sciences students. Educ Health (Abingdon).

[34] Lidskog M, Lofmark A, Ahlstrom G (2008). Learning about each other: Students' conceptions before and after interprofessional education on a training ward. J Interprof Care.

[35] Ateah CA, Snow W, Wener P, MacDonald L, Metge C, Davis P, Fricke M, Ludwig S, Anderson J (2011). Stereotyping as a barrier to collaboration: does interprofessional education make a difference?. Nurse Educ Today.

[36] Norgaard B, Draborg E, Vestergaard E, Odgaard E, Jensen DC, Sorensen J (2013). Interprofessional clinical training improves self-efficacy of health care students. Med Teach.

[37] Hood K, Cant R, Leech M, Baulch J, Gilbee A (2014). Trying on the professional self: nursing students' perceptions of learning about roles, identity and teamwork in an interprofessional clinical placement. Appl Nurs Res.

[38] Jacobsen F, Lindqvist S (2009). A two-week stay in an Interprofessional training unit changes students' attitudes to health professionals. J Interprof Care.

[39] Brock D, Abu-Rish E, Chiu CR, Hammer D, Wilson S, Vorvick L, Blondon K, Schaad D, Liner D, Zierler B (2013). Interprofessional education in team communication: working together to improve patient safety. BMJ Qual Saf.

[40] Kyrkjebo JM, Brattebo G, Smith-Strom H (2006). Improving patient safety by using interprofessional simulation training in health professional education. J Interprof Care.

[41] Hylin U, Lonka K, Ponzer S (2011). Students' approaches to learning in clinical interprofessional context. Med Teach.

[42] Hylin U, Nyholm H, Mattiasson AC, Ponzer S (2007). Interprofessional training in clinical practice on a training ward for healthcare students: a two-year follow-up. J Interprof Care.

[43] Jakobsen F, Larsen K, Hansen TB (2010). This is the closest I have come to being compared to a doctor: views of medical students on clinical clerkship in an Interprofessional training unit. Med Teach.

[44] van Soeren M, Devlin-Cop S, MacMillan K, Baker L, Egan-Lee E, Reeves S (2011). Simulated interprofessional education: an analysis of teaching and learning processes. J Interprof Care.

[45] Hutchings M, Scammell J, Quinney A (2013). Praxis and reflexivity for interprofessional education: towards an inclusive theoretical framework for learning. J Interprof Care.

[46] Ruebling I, Pole D, Breitbach AP, Frager A, Kettenbach G, Westhus N, Kienstra K, Carlson J (2013). A comparison of student attitudes and perceptions before and after an introductory interprofessional education experience. J Interprof Care.

[47] Horsburgh M, Lamdin R, Williamson E (2001). Multiprofessional learning: the attitudes of medical, nursing and pharmacy students to shared learning. Med Educ.

[48] Hayashi T, Shinozaki H, Makino T, Ogawara H, Asakawa Y, Iwasaki K, Matsuda T, Abe Y, Tozato F, Koizumi M (2012). Changes in attitudes toward interprofessional health care teams and education in the first- and third-year undergraduate students. J Interprof Care.

[49] Kururi N, Makino T, Kazama H, Tokita Y, Matsui H, Lee B, Kanaizumi S, Abe Y, Uchida Y, Asakawa Y (2014). Repeated cross-sectional study of the longitudinal changes in attitudes toward interprofessional health care teams amongst undergraduate students. J Interprof Care.

[50] Baker C, Pulling C, McGraw R, Dagnone JD, Hopkins-Rosseel D, Medves J (2008). Simulation in interprofessional education for patient-centred collaborative care. J Adv Nurs.

[51] Rosenfield D, Oandasan I, Reeves S (2011). Perceptions versus reality: a qualitative study of students' expectations and experiences of interprofessional education. Med Educ.

[52] Tofil NM, Morris JL, Peterson DT, Watts P, Epps C, Harrington KF, Leon K, Pierce C, White ML (2014). Interprofessional simulation training improves knowledge and teamwork in nursing and medical students during internal medicine clerkship. J Hosp Med.

[53] Nisbet G, Hendry GD, Rolls G, Field MJ (2008). Interprofessional learning for pre-qualification health care students: an outcomes-based evaluation. J Interprof Care.

[54] Freeth D, Reeves S, Goreham C, Parker P, Haynes S, Pearson S (2001). 'Real life' clinical learning on an interprofessional training ward. Nurse Educ Today.

[55] Cooper H, Spencer-Dawe E, McLean E (2005). Beginning the process of teamwork: design, implementation and evaluation of an inter-professional education intervention for first year undergraduate students. J Interprof Care.

[56] Siassakos D, Timmons C, Hogg F, Epee M, Marshall L, Draycott T (2009). Evaluation of a strategy to improve undergraduate experience in obstetrics and gynaecology. Med Educ.

[57] Hobgood C, Sherwood G, Frush K, Hollar D, Maynard L, Foster B, Sawning S, Woodyard D, Durham C, Wright M (2010). Teamwork training with nursing and medical students: does the method matter? Results of an interinstitutional, interdisciplinary collaboration. Qual Saf Health Care.

[58] Blue AV, Charles L, Howell D, Koutalos Y, Mitcham M, Nappi J, Zoller J (2010). Introducing students to patient safety through an online interprofessional course. Adv Med Educ Pract.

[59] McCaffrey R, Hayes R, Cassell A, Miller-Reyes S, Donaldson A, Ferrell C (2012). The effect of an educational programme on attitudes of nurses and medical residents towards the benefits of positive communication and collaboration. J Adv Nurs.

[60] Lidskog M, Lofmark A, Ahlstrom G (2009). Learning through participating on an interprofessional training ward. J Interprof Care.

[61] Tunstall-Pedoe S, Rink E, Hilton S (2003). Student attitudes to undergraduate interprofessional education. J Interprof Care.

[62] Hood K, Cant R, Baulch J, Gilbee A, Leech M, Anderson A, Davies K (2014). Prior experience of interprofessional learning enhances undergraduate nursing and healthcare students' professional identity and attitudes to teamwork. Nurse Educ Pract.

[63] Hansson A, Foldevi M, Mattsson B (2010). Medical students' attitudes toward collaboration between doctors and nurses : a comparison between two Swedish universities. J Interprof Care.

[64] Carpenter J (1995). Doctors and nurses: stereotypes and stereotype change in interprofessional education. J Interprof Care.

[65] Anderson ES, Thorpe LN (2008). Early interprofessional interactions: does student age matter?. J Interprof Care.

[66] Baggs JG, Schmitt MH (1997). Nurses' and resident physicians' perceptions of the process of collaboration in an MICU. Res Nurs Health.

[67] McGrail KA, Morse DS, Glessner T, Gardner K (2009). "what is found there?": qualitative analysis of physician-nurse collaboration stories. J Gen Intern Med.

[68] Ponzer S, Hylin U, Kusoffsky A, Lauffs M, Lonka K, Mattiasson A, Nordström G (2004). Interprofessional training in the context of clinical practice: goals and students' perceptions on clinical education wards. Med Educ.

[69] Hallin K, Kiessling A, Waldner A, Henriksson P (2009). Active interprofessional education in a patient based setting increases perceived collaborative and professional competence. Med Teach.

[70] Laschinger HK, Weston W (1995). Role perceptions of freshman and senior nursing and medical students and attitudes toward collaborative decision making. J Prof Nurs.

[71] Lachmann H, Ponzer S, Johansson UB, Benson L, Karlgren K (2013). Capturing students' learning experiences and academic emotions at an interprofessional training ward. J Interprof Care.

[72] Lindblom P, Scheja M, Torell E, Astrand P, Fellännder-Tsai L (2007). Learning orthopaedics: assessing medical students' experiences of interprofessional training in an orthopaedic clinical education ward. J Interprof Care.

[73] Wijma MB (1999). Student attitudes towards the goals of an inter-professional training ward. Med Teach.

[74] Brewer ML, Stewart-Wynne EG (2013). An Australian hospital-based student training ward delivering safe, client-centred care while developing students' interprofessional practice capabilities. J Interprof Care.

[75] Falk AL, Hult H, Hammar M, Hopwood N, Dahlgren MA (2013). One site fits all? A student ward as a learning practice for interprofessional development. J Interprof Care.

[76] Ericson A, Masiello I, Bolinder G (2012). Interprofessional clinical training for undergraduate students in an emergency department setting. J Interprof Care.

[77] Jacobsen F, Fink AM, Marcussen V, Larsen K, Hansen TB (2009). Interprofessional undergraduate clinical learning: results from a three year project in a Danish Interprofessional training unit. J Interprof Care.

[78] Curran VR, Sharpe D, Flynn K, Button P (2010). A longitudinal study of the effect of an interprofessional education curriculum on student satisfaction and attitudes towards interprofessional teamwork and education. J Interprof Care.

[79] Lidskog M, Lofmark A, Ahlstrom G (2007). Interprofessional education on a training ward for older people: students' conceptions of nurses, occupational therapists and social workers. J Interprof Care.

[80] MacDonnell CP, Rege SV, Misto K, Dollase R, George P (2012). An introductory interprofessional exercise for healthcare students. Am J Pharm Educ.

[81] Gillan C, Lovrics E, Halpern E, Wiljer D, Harnett N (2011). The evaluation of learner outcomes in interprofessional continuing education: a literature review and an analysis of survey instruments. Med Teach.

[82] Thistlethwaite J, Moran M (2010). Learning outcomes for interprofessional education (IPE): literature review and synthesis. J Interprof Care.

[83] Barr H, Koppel I, Reeves S, Hammick M, Freeth DS (2008). Effective interprofessional education: argument, assumption and evidence (promoting Partnership for Health).

[84] Thistlethwaite J, Kumar K, Moran M, Saunders R, Carr S: An exploratory review of pre-qualification interprofessional education evaluations. J Interprof Care 2014(0):1-6. 10.3109/13561820.2014.985292 25431833

[85] Hind M, Norman I, Cooper S, Gill E, Hilton R, Judd P, Jones SC (2003). Interprofessional perceptions of health care students. J Interprof Care.

[86] ten Cate O, Snell L, Mann K, Vermunt J (2004). Orienting teaching toward the learning process. Acad Med.

[87] Hacker DJ, Dunlosky J, Graesser AC (2009). Handbook of metacognition in education.

[88] Crooks T (2015). The impact of classroom evaluation practices on students. Rev Educ Res.

[89] Artino AR, La Rochelle JS, Durning SJ (2010). Second-year medical students' motivational beliefs, emotions, and achievement. Med Educ.

[90] Oandasan I, Reeves SC (2005). Key elements for interprofessional education. Part 1: the learner, the educator and the learning context. J Interprof Care.

[91] Gallé J, Lingard L (2010). A medical student's perspective of participation in an interprofessional education placement: an autoethnography. J Interprof Care.

