# Supplementary material for: Perceptions of residents, medical and nursing students about Interprofessional education: a systematic review of the quantitative and qualitative literature
Source: BMC Med Educ. 2017 May 3;17:77. doi: 10.1186/s12909-017-0909-0 (PMC5415777; doi:10.1186/s12909-017-0909-0)
Supplement: Additional file 1: — Table of papers included in the review. For each included paper the study reference, country, setting, number of students, research objectives, findings relevant to the review, type of study and pedagogical approach are provided. (DOC 226 kb) [file 12909_2017_909_MOESM1_ESM.doc]

**Additional File 1**

**Table of papers included in the review**

| **Study Reference** | **Country, Setting,**  **N** | **Research Objectives** | **Findings relevant to the review** | **Type of study** | **Pedagogical**  **approach** |
| --- | --- | --- | --- | --- | --- |
| E.S. Anderson & L.N. Thorpe (2008)  Early interprofessional interactions: Does student  age matter?  J Interprof Care, June; 22(3): 263 – 282 | Leicester, UK  N= 207 nurs stud  65 med graduates  235 med undergrad.  81 in Focus group | Investigation of students’ perceptions about their readiness for teamwork following an IPL intervention. | Younger students achieved more learning outcomes as compared to older students (>25 years) and were more satisfied with the intervention.  Undergraduate medical, nursing, social work and pharmacy students achieved more learning outcomes than graduate medical and social work students, i.e. the readiness was dependent on the profession and seniority. (individual level). | QT: RIPLS pre and  post  QL   - free answer - Focus groups | PBL, SG, A  Gillan KP2a |
| Ateah CA, Snow W, Wener P, MacDonald L, Metge C, Davis P, Fricke M, Ludwig S, Anderson J.  (2011)  Stereotyping as a barrier to collaboration: Does Interprofessional education make a difference?  Nurse Educ Today. Feb;31(2):208-13 | Canada, Winnipeg  Workplace setting  N:  Control= 16  Intervention= 17  Immersion= 18 | Do the attributes of 7 health professions recorded at the beginning of the study change by IPE or practice immersions.  Two types of interventions: in classroom IP discussions, in IPC practice in small IP student groups. | Learning together in an interprofessional environment had a positive effect on the perceptions of health care students about interpersonal skills, professional competence, leadership, academic ability, being a team player, being an independent worker, confidence, decision-making and practical skills of the other health professions. This IPE increases the readiness of students for IPC. | Mixed methods, SSRQ | SG, Shadowing of IP member, A  Thistlethwaite KP 2a |
| Judith Baggs & Madeline H. Schmitt (1997)  Nurses’ and Resident Physicians’ Perceptions  of the Process of Collaboration in an MICU  Research in Nursing & Health, 20, 71–80 | USA, Rochester  N = 5 1^st^ yr + 5 2^nd^ yr residents and  10 nurses MICU | Qualitative study to compare perceptions of nurses and residents of the process of nurse–physician collaboration, using grounded theory method for concept development. | Facilitators of IPC at individual level – Being available, being receptive: conveying respect, interest and trust.  Barriers of IPC at individual level – arrogance or disinterest, aggressive behavior, nurses delaying residents’ orders, because they feel that they are the patients’ protectors.  Barriers of IPC at the process level – lack of time. | QL: semi structured, open ended questions from interview guide. |  |

| Baker C, Pulling C, McGraw R, Dagnone JD, et al. (2008)  Simulation in interprofessional education for patient−centred collaborative care  J Adv Nurs., Nov;64(4):372-9. | Canada, Ontario  1^st^ group=  101 Nurs, 42 Med  70 junior resid.  2^nd^ group=  45 Nurs, 20 Med  7 junior residents | The reaction of learners and teachers to IPE and simulation. | Students scored high on understanding of team roles and team interdependence after a simulation-based IPE intervention. | QT: Questionnaire open ended+ rating  2^nd^ group:  IEPS | PBL, simulation patient, SG, A  Gillan KP2a |
| --- | --- | --- | --- | --- | --- |
| Amy V Blue, Laurine Charles  David Howell, Yiannis Koutalos  Maralynne Mitcham, Jean Nappi, James Zoller (2010)  Introducing students to patient safety through an online interprofessional course  Advances in Medical Education and Practice:1 107–114 | USA, Charleston  N= 267 (91%) students | Small group online course with assessment through final inclass presentation.  Students worked in IP groups on a project requiring them to complete a root cause analysis and develop recommendations based on a sentinel event case. | **F =** students indicated that face-to-face time would be an improvement. They enjoyed working with students from other professions (=F process)  Readiness increased as appreciation of IP collaboration increased and knowledge of the roles of own and other professions increased.  Students were asked to complete a paper-and-pencil end of- course evaluation form at the last class session. | Evaluation items addressed broad and specific course learning goals, including course content areas. | PBL, simulation patient, A,  SG online |
| Bradley P, Cooper S, Duncan F.  (2009)  A mixed-methods study of interprofessional learning of resuscitation skills  Medical Education 43: 912–922 | UK, Plymouth  2^nd^ yr students: 170 Med, 45 Nurs  at random in uni-professional group or IP group | To identify the effects of interprofessional teaching resuscitation skills on medical and nursing students’ attitudes, leadership, team-working and performance skills. | No significant difference between IP and uni-professional teams for leadership, team dynamics or resuscitation tasks performance. Gender, previous IP learning, professional background and previous leadership experience had no significant effect. Barriers to IPE at individual level = Uncertainty about roles in the IP environment, Concerns about different levels of learning, Hesitancy. At process level: Lectures as a teaching method, Timetabling issues. At cultural level – Hierarchy, Stereotypical views of doctors. RIPLS subscales for professional identity and team-working increased significantly post-intervention for IP groups but returned to pre-test levels by 3–4 months. | QL:  video observation and focus group interview on opinions and attitudes towards IPE  QT: RIPLS | Thistlethwaite KP 1  Gillan KP2a |
| Douglas Brock, Erin Abu-Rish, Chia-Ru Chiu, Dana Hammer,  Sharon Wilson, Linda Vorvick, Katherine Blondon, Douglas Schaad, Debra Liner, Brenda Zierler (2013)  Interprofessional education in team communication:  working together to improve patient safety  BMJ Qual Saf;22:414–423. | USA, Seattle  N= 149 students  73 Med (33 male)  46 Nurs (5 male)  30 other stud  In focal areas Obstetrics, Paediatrics or Adult care | Investigation whether training in team communication skills leads to increased readiness through increased self-efficacy, motivation etc. towards IPC. | Training in team communication skills acts as a **F** by increasing motivation, positive attitude towards IPC and perception of utility of IPE.  F (process) - Students valued most - p.420:  Opportunity to work with students from different professional schools; learning and practicing specific communication skills in a supportive environment; practicing skills within an IP team. | QT  Pre and post-intervention: TeamSTEPPS  Teamwork Attitudes Questionnaire (TAQ).  Attitudes were assessed by  ‘AMUSE.’ |  |
| Margo L. Brewer & Edward G. Stewart-Wynne (2013)  An Australian hospital-based student training ward delivering safe, client-centred care while developing students’ interprofessional practice capabilities  J Interprof Care, 27:6, 482-488, DOI:  10.3109/13561820.2013.811639 | Australia, Perth  Med  Nurs  OT  PT  And students from 4 other professions | To investigate whether a SLW is sufficient to establish IPP capabilities in final year students, assessed by qualified professionals who observed the students for 75 hrs during 2-3 weeks. | Interprofessional Capability Assessment Tool:  At the end of the clerkship, the majority of students were judged by the interprofessional facilitators to be 3 or 4 on the grading rubric for communication, professionalism, collaborative practice and client-centred values, which is high.  The workload was perceived as high, which might account for the fact that 76 of 79 students did not complete both the pre and post IP Socialization and Valuing scale. The placement was rated as ‘good’ by 38% and ‘excellent’ by 41 % of respondents.  Open ended questions:  Placement led to a clearer understanding of the roles, responsibilities and capabilities of other professions.  F = The great level of responsibility and autonomy in combination with approachable and supportive facilitation was perceived as a valuable experience.  75% of Clients completed the satisfaction survey, and rated the SLW with a median of ‘highly satisfied’. | Mixed methods:   - Curtin Univ. Interprofessional Capability Assessment Tool (4 point scale) - IP Socializa-tion and Valuing scale (King, et al. 2010) - Open ended questions - Client satisfaction questionnaire   Assessment by facilitator using a rubric and for reflection by students | SLW |
| John Carpenter (1995)  Doctors and Nurses: Stereotypes and Stereotype  Change in Interprofessional Education  J Interprof Care, 9:2, 151-161, DOI:  10.3109/13561829509047849 | UK, University of Kent  Students in their final yr.: 16 nurse (all female)  23 medical (18 females) | A list of stereotypes was generated by letting a mixed group of students brainstorm about characteristics of professions. This list was used to rate the background perceptions of 39 students pre and post an IPE during 1 yr. | - Status in society: for doctors rated higher than nurses, by both groups but interestingly the medics rated the status of nurses a little higher than did the nursing students their own. - Professional competence: both groups respected the other groups competence, but their own group rated slightly higher. - **B Cultural =** Perceived hetero-stereotypes –what one group believes that other held over them: both groups are accurate in their perception of the other groups’ views.   Medical students   - Identification with own profession: significantly stronger than nurses - Auto-stereotypes – what each group holds about the own profession. Doctors were seen as confident, dedicated and caring (mean rating 5.0 and above) - Hetero-stereotypes: medical stud considered nurses to be caring, dedicated and moderately good communicators, with a tendency to be ‘do-gooders’ - Changes after IPE: med stud did not change their (stereotyped) view of nurses.   Nursing students   - Identification with own profession: not as high as medical students at this stage of their education. - Auto-stereotypes – caring, dedicated and good communicators and rejection of the arrogant, detached or dithering characterisation. - Hetero-stereotypes – considered doctors to be dedicated, confident and decisive, but also detached, arrogant and poor communicators. - F = Changes after the IPE: nurses saw doctors as less detached, more caring and better communicators. | QL to develop a questionnaire regarding characteristics of professions  QT with that questionnaire | SCL  Thistlethwaite & Moran  KP 2b  Gillan KP2a |
| Helen Cooper, Eileen Spencer-Dawe & Elspeth Mclean (2005)  Beginning the process of teamwork: Design,  implementation and evaluation of an inter-professional  education intervention for first year undergraduate  students  J Interprof Care, October; 19(5): 492 – 508 | UK, Liverpool  N= 237 stud | Intervention included a staff-training programme, e-learning materials and interprofessional team working skills workshops in academic environment to promote theoretical learning about team working, raising awareness about collaborative practice, and its link to improving the effectiveness of care delivery . | Qualitative data showed that IPE served to increase students’ confidence in their own professional identity and F = helped them to value difference, making them better prepared for clinical placement. | QL  FG 21 stud + written reflection after each workshop  +  QT  RIPLS pre + post | SCL  Thistlethwaite & Moran  KP 2b  Gillan KP2a |
| Samantha Coster, Ian Norman, Trevor Murrells, Sheila Kitchen, Elizabeth Meerabeau, Enkanah Sooboodoo , Lynda d’Avray (2008) Interprofessional attitudes amongst undergraduate students in the health professions: A longitudinal questionnaire survey  Internat. J. of Nursing Studies 45; 1667–1681 | UK  N = 1683 pre-registration students from  8 healthcare groups from 3 higher education institutions. | To measure changes in RIPL, professional identification and amount of contact between students of different professional groups.  To examine the influence of professional group, student characteristics and an IPE course on these scores over time. | Readiness for IPE and professional identity were highest at entry and declined significantly over time. Females were better on both than males.  Being African was associated with higher readiness and being Asian with lower readiness.  Having earlier health care experience was associated with higher readiness.  A small but significant positive relationship between professional identity and readiness for IPL was maintained over time.  B = Females stronger professional identification than males and higher RIPLS. | QT: Longitudinal panel questionn. survey at 4 time-points  RIPLS  IPL/IPE eval.  Professional Identity Scale (Brown, 1986) | Gillan KP2a |

| Curran, Vernon R.; Mugford, J. Gerry; Law, Rebecca M. T.; MacDonald, Sandra (2005)  Influence of an interprofessional HIV/AIDS education program on role perception, attitudes and teamwork skills of undergraduate health sciences students Education for Health: Change in Learning & Practice, Vol 18(1), Mar; 32-44 | Canada,  Newfoundland  45 3^rd^yr Nurs stud  62 2^nd^yr Med stud  26 final yr Pharm stud  Small groups (8-10) with tutor | To assess the changes in role perception, attitudes toward collaboration, self-reported teamwork skills and satisfaction with a Shared learning experience of an undergraduate paper based case-study + simulation patient contact. Combined 1 group pre+ post test + time series study design. | Readiness for IPC increased after IPE intervention through increased understanding of others’ professional roles and increased perceived competence in IPC.  F = Standardized patients were effective in fostering an experience of realism and motivating collaboration between students. | QT Role Perception Checklist + Weekly Team Inventory  QL (Observations) | PBL standardized patient |
| --- | --- | --- | --- | --- | --- |
| Curran VR, Sharpe D, Flynn K, Button P. (2010)  A longitudinal study of the effect of an interprofessional education curriculum on student satisfaction and attitudes towards interprofessional teamwork and education  J Interprof Care. Jan;24(1):41-52 | Canada, Newfoundland  2078 Nursing students  1301 Medical students (+ 2 other groups) | To examine the longitudinal effects of a new IPE and the effect on attitudes toward IP teamwork and toward IPE and evaluate students satisfaction with the IPE model. | F = Panel discussion of practitioners of IP-teams; Interacting with Simulated patient; Satisfaction to meet students and learn about their roles during face to face small group learning activities.  B = Online small group discussion being redundant in combination with face to face small group learning activity; scheduling; not enough diverse students in their small learning group. | QT based on ATHCTS, ATIPE based on RIPLS +  Interacting with Simulation Patient; 2 open ended questions  QL: 619 comments. | PBL  Gillan KP2a  Thistlethwaite KP 2a |

| Dale J. DeMatteo and Scott Reeves (2013)  Introducing first year students to interprofessionalism: Exploring professional identity in the “enterprise culture”: A Foucauldian analysis  J Interprof Care 27: 27–33 | Canada, Toronto  N= 30 students from 5 professions incl. med, not nurs. | Exploration of the experiences of, and thoughts on, IPE and care of first year health science students at a large Canadian university within a broad socioeconomic context. | F (at process level) = Taking personal responsibility for “saving health care” through IPE  F (at individual level) = Perception of IPE as a tool of efficiency and excellence.  B (at individual level = The need to “sell oneself” to other professions,  B= Shifting professional/ client relations (at cultural level),  B individual level = finding one’s way in the enterprise clinic. | QL: Focus groups | Thistlethwaitesecondary analysis |
| --- | --- | --- | --- | --- | --- |
| Anne Ericson, Italo Masiello & Gunilla Bolinder (2012)  Interprofessional  clinical training for undergraduate students in an emergency department setting  Journal of Interprofessional Care, 26:4, 319-325, DOI: 10.3109/13561820.2012.676109 | Sweden, Stockholm  75% of total students in an emergency ward:  Med 89 (64%)  Nurse 102 (78%)  PT 43 (75%)  Mean age all categories between 27-29 yrs (spread 23 – 39) | Describing the organizational and educational model for the training in the ward and comparing the attitudes of the participating students | QT: Pre and post questionnaire showed self- perceived increase of knowledge and understanding of own professional role, and professional role of others for all students categories.  QL: Positive statements were 52%, concerning Team training (34%), having responsibility and independence (22% ) and learning about one’s own and other professional role (16%) and about supervisors (16%). Rest was miscellaneous.  Negative statements formed 27%, of which were about changes of supervision for the nurses during the two weeks (41%) or no supervision in the evenings for the medical students. Suggestions for improvement were diverse. | QT: questionnaire validated by Ponzer (2004), with two additional questions  QL: answers to open ended questions, 883 statements. |  |
| Annika Lindh Falk, Håkan Hult, Mats Hammar, Nick Hopwood & Madeleine Abrandt Dahlgren (2013)  One site fits all?  A student ward as a learning  practice for interprofessional development  J Interprof Care, 27:6, 476-481, DOI:  10.3109/13561820.2013.807224 | Sweden, Linköping  Med  Nurs  OT  PT | The theoretical lens of practice theory (the doings and sayings of people in the context and in relationships) is used to understand how students perceive collaboration and learning in an Interprofessional training ward (IPTW). From these insights the researchers want to better understand the IPTW learning and practice. | Medical students experienced the ward rounds (round table discussion by the team) as an opportunity to interact with other students using their specific medical expertise.  Nursing students considered the organizational and administrative planning of the daily work an important aspect of learning their professional responsibilities.  OT and PT students could bring their professional opinion of specific patient situations to the team – a powerful learning experience.  Some students objected to the general caring tasks required from them – they considered it an unexpected situation. It was intentional, to create shared understandings across professions. Other students saw it as vital in establishing trust among the team.  Medical students were not used to the requirement to be present in the ward all day. | QT: adapted evaluation form with 3 domains: conditions for learning and collaboration; professional development; general experiences and valuing of the IPTW.  QL: 12 open ended questions regarding experienced stress, time constraints, dealing with ethical situations, the role of reflection for learning etc. | A  SLW |
| M.B. Fallsberg, K. Wijma (1999)  Student attitudes towards the  goals of an inter-professional training ward  Medical Teacher, 21:6, 576-581, DOI: 10.1080/01421599978997 | Sweden, Linköping  Med Lab tech. 11  Med 34  Nursing 74  OT 23  PT 21 | Would participation lead to an increase in scores on statements about 4 characteristics of the SLW:   1. Opportunity to collaborate with all professions 2. Development of own professional role 3. An understanding of the skills, attitudes and competencies of other professions 4. Recognition of the needs of patients. | During the two weeks the within group scores on the statements showed practically no difference. All groups agreed with all statements, but not to the same extent regarding a-d:   1. Med Lab Techn scored lower than the other groups; 2. Med Lab techn and Medial students were not positive. In contrast Nursing, OT and PT were positive. 3. This hypothesis was confirmed 4. The Medical group was less positive than the other groups. | QT | SLW |
| Della Freeth, Scott Reeves, Celia Goreham, Pam Parker,  Sallyann Haynes and Sara Pearson(2001)  ‘Real life’ clinical learning on  an interprofessional training  ward  Nurse Education Today 21, 366–372 | UK, London  Facilitators  (*n* = 10), clinical staff (*n* = 13); Students (*n* = 36). | Multi-method evaluation of a two week interprofessional training ward placement for medical, nursing, occupational therapy and physiotherapy students. | Nursing students perceptions after an IPE intervention. F = Real life experiences (Authentic), Team reflection meetings, wearing a uniform which pegged all the IP team members at the same level. Team duties, the way in which they were arranged in this intervention, were perceived negatively. | QT  QL: Focusgroups | PBL, SG, A  SLW |
| Conor Gilligan, Sue Outram and Tracy Levett-Jones (2014)  Recommendations from recent graduates in medicine, nursing and pharmacy on improving  interprofessional education in university programs: a qualitative study  BMC Medical Education 14:52 | Australia, NSW  68 newly graduates:  28 Nurs, 17 Med and 23 Pharm. | New graduates’ reflections on their experiences of IPE as  part of their university degree, as well as recommendations to improve IPE before graduation. They were unanimous in valuing IPE from their current perspective of being in the health workforce. | F for IPE (at process level) = Active engagement with other professions.  B = Little social contact with students from the other professions, creating professional silos (at process level).  Dissonance between stated faculty values and educational practice (B at culture/organizational level). | QL: Focus groups |  |
| Karin Hallin, Anna Kiessling, Annika Waldner & Peter Henriksson (2009)  Active interprofessional education in a patient  based setting increases perceived collaborative  and professional competence  Medical Teacher, 31:2, 151-157, DOI:  10.1080/01421590802216258 | Sweden, Stockholm  N= 616, in all groups except Medial mostly female.  Med  Nurs  OT  PT | Replication of research by Ponzer (2004), with pre and post questionnaires.  This study investigated how students perceived their professional and Interprofessional training. | Medical students perceived to have gained significantly more knowledge of OT and PT than from Nursing.  Nursing students perceived to have gained significantly more knowledge of OT and PT than from the medical profession.  All students groups reported a gain in understanding of the importance of communication and teamwork in patient care, with the medical students reporting the greatest gain and they also gained most in clarity of own professional role. All groups perceived that the teamwork had resulted in meeting the patients’ needs and insight in the importance of professional competence. | QT (pre and post questionnaire) | A: stimulating students to take an active part in the teamwork of the ward.  SLW |
| Hansson A, Foldevi M, Mattsson B. (2010)  Medical students' attitudes toward collaboration between doctors and nurses - a comparison between two Swedish universities.  J Interprof Care. May; 24(3):242-50 | Sweden  84+50 1^st^ yr  Med stud  75+51 final yr Med stud | To investigate differences in attitudes toward collaboration between doctors and nurses among medical students in first and final year. | Readiness for IPC was higher in females than males and in first year students than fourth year students. There was no effect of IPC intervention on the readiness for IPC. | QT  Jefferson Scale of Attitudes toward Phys.-N collaboration  IPL/IPE evaluation | Gillan KP2a |
| Tomoko Hayashi, Hiromitsu Shinozaki, Takatoshi Makino et al. (2012)  Changes in attitudes toward interprofessional health care teams and education in the first- and third-year undergraduate students  J Interprof Care; 26: 100–107 | Japan, Maebashi  N= 1st yr  3rd yr groups consisting of 80 Nurs, 40 Lab, 20 PT and 20 OT | To compare two styles of IPE delivery for nursing, physical therapy, occupational therapy, laboratory sciences students. | Readiness for IPE and attitude towards health care teams improved after IPE involving teaching through practice (F at Process level) and decreased after IPE involving teaching through lectures. (Barrier at Process level). | QT:  ATHCTS  and  the modified RIPLS (two subscales). | SCL , SG, A  Gillan KP2a  Thistlethwaite KP 2a |
| Cherri Hobgood, Gwen Sherwood et al. on behalf of the Interprofessional Patient Safety Education Collaborative (2010)  Teamwork training with nursing and medical students: does the method matter?  Results of an Inter institutional, interdisciplinary collaboration.  Qual Saf Health Care;19:e25. | USA, North Carolina  N=  203 senior Nurs stud  235 Med stud 4^th^ yr | To investigate the effectiveness of 4 types of instructional methods for IPE: lecture, audience response didactic, role play and high fidelity simulation. | Knowledge, attitude and skills all showed a significant change after any of the IPE interventions.  All were patient problem centred simulations. The readiness for IPC increased with all these four types of instructional methods. | QT: 36-item teamwork attitudes (CHIRP), 12-it. teamwork knowledge test,  and modified 20-item Mayo High Performance  Teamwork Scale (MHPTS). | PBL, A |
| Kerry Hood, Robyn Cant, Julie Baulch et al. (2014)  Prior experience of interprofessional learning enhances undergraduate nursing and healthcare students’ professional identity  and attitudes to teamwork.  Nurse Education in Practice 14; 117-122 | Australia, Clayton  N= 741 students, response rate approx. 46% , of medicine, nursing, dietetics, midwifery, PT, nurse-paramedic, scheduled for clinical placement. | Survey conducted on medical, nursing, midwifery, nursing emergency health and dietetics students. | Readiness for IPL was high in year 3, decreased in year 4 and increased again in year 5. Nursing students had more positive attitudes toward team collaboration, shared learning and recognized everyone’s role in a health care system. F individual = Students with prior experience of IPL had more readiness for IPL. Females had stronger negative professional identity which meant that they were not in favor of professional learning and they had less positive attitude toward roles and responsibilities. Barriers for IPE at individual level: Year 4 of study, Being a female. | QT: RIPLS | Gillan KP2a |
| Kerry Hood, Robyn Cant et al. (2014)  Trying on the professional self: nursing students' perceptions of learning about roles, identity and teamwork in an interprofessional  clinical placement  Applied Nursing Research 27; 109–114 | Australia, Clayton  N= 10 Nursing and 9 medical students | To describe how senior nursing students viewed the clinical learning environment and  matured their professional identity through IP learning in a student-led hospital ‘ward’. | Students who went through a ward-based interprofessional clinical learning initiative perceived autonomy in their learning and work, better understanding of their own and other professional roles, better ways of communicating and collaborating and becoming a functioning team. Thus IPE enhanced their readiness for IPC. | Mixed Methods  QT: IP Clinical Placement Learning Environment Inventory  Focus groups | PBL  SLW |
| Margaret Horsburgh, Rain Lamdin & Emma Williamson  (2001)  Multiprofessional learning: the attitudes of medical, nursing  and pharmacy students to shared learning  Medical Education;35:876 - 883 | New Zealand, Auckland  1^st^ yr students:  Med 79,  Nurs 49,  Pharm 52 | To quantify the attitudes of 1^st^ year students toward IPL at course commence.  First-year medical, nursing and pharmacy students considered shared learning to enhance their effectiveness at work. | Medical students ( lower readiness for IPL) were less sure of their professional roles than nursing and pharmacy students.  Males were more sure of their professional role than females and demonstrated a greater readiness for IPL. | QT  RIPLS  IPL | Gillan KP2a |
| Maggie Hutchings, Janet Scammell and Anne Quinney  (2013)  Praxis and reflexivity for interprofessional education: towards an inclusive theoretical framework for learning  J Interprof Care 27(5): 358–366 | UK, Bournemouth  2006 N= 252  (61% Nurs students)  2007 N= 143  (57% Nurs stud) | To make a case for a theoretical model for IPE focusing on the dynamic relationships between the F and B at work in IPE, bridging the gap between individual and social structural orders of practice to enable transformative learning. | B for IPE= Differences in technological savviness of students (at process level),  F= A move from shared teaching to collaborative learning,  F= Value in learning about professional differences and identity (at individual level). | QL: Focus groups and QT when groups could not be scheduled for Focus group | PBL |
| Uffe Hylin, Helena Nyholm, Anne-Cathrine Mattiasson,  & Sari Ponzer (2007)  Interprofessional training in clinical practice on a training ward for healthcare students: A two-year follow-up  J Interprof Care June; 21(3):  277 – 288 | Sweden, Stockholm  N = 283 students  60 Med;  151 Nurs;  72 OT+ PT. | To investigate lasting impressions of a 2 week ITU regarding understanding roles of other HCP and the importance of communication for teamwork while also training profession specific aspects. | Professional role development was both enhanced - better understanding of own and other’s professional roles - and hampered because of few profession-specific tasks. Teamwork bred the ground for future collaboration, but difficulties in IPC were witnessed. Profession-specific tutoring (nurse-nursing student) was liked by some and disliked by other students. Most students appreciated the opportunity to participate in nursing care, but some found that it diminished their professional role. These factors could be both F and B at process level for IPE. | QT  QL: open ended questions | SLW |
| Uffe Hylin, Kirsti Lonka & Sari Ponzer (2011)  Students’ approaches to learning in clinical  interprofessional context  Medical Teacher; 33: e204–e210 | Sweden, Stockholm  369 stud including 192 nurs | Investigation of health care students’ evaluations of interprofessional clinical training in relation to their study orientations. | Approaches to learning can be a facilitator or barrier for IPE. Low collaborative approach can be a barrier as these students give lower importance to IPC and are less satisfied with it.  Approaches to learning do not affect the growth of own professional role and the understanding of professional roles of others after IPE. | QT 19 items of the Conceptions of Learning and Knowledge Questionnaire, 3 subscales. | PBL |
| Flemming Jacobson & Susanne Lindqvist (2009)  A two-week stay in an Interprofessional Training Unit changes students’ attitudes to health professionals  J Interprof Care, May; 23(3): 242–250 | Denmark Holstebro & Aarhus  N = 169, students from  Nurs 69  OT 29, PT 31  Med 33 | To evaluate the students’ learning experience in an ITU. It also shows students’ attitudes of their own and other professions, both at the outset and at the end of their time at the ITU. | After undergoing Interprofessional Training Unit as an IPE initiative students from all professions had a more positive perception of the other professions about their “subservient” (items: valuing autonomy, assertiveness and technical focus) and “caring” behaviour (items regarding empathy, approachability and teamwork). This could increase the readiness for IPC. So, ITU as an IPE initiative can be a Facilitator for IPC. | QT: pre and post ITU  AHPQ (Att toward Health Professions Questionnaire – Lindqvist, 2005) | PBL patient centred  SLW  Thistlethwaite KP 2a |
| Flemming Jacobsen, Anna Marie Fink, Vibeke Marcussen,  Kristian Larsen, & Torben Bæk Hansen (2009)  Interprofessional undergraduate clinical learning: Results from a three year project in a Danish Interprofessional Training Unit  J Interprof Care, 2009; 23(1): 30–40 | nursing, physiotherapy, occupational therapy and  medicine | This study investigated the fulfilment of goals of the ITU as evaluated by  students, managers, deans, clinical tutors and others with an interest in the ITU. The goals  of the ITU are to teach the students:  . IP teamwork;  . strengthening of their individual professional  roles (Uniprofessional).  . about working in an organization where several professions  work closely together on giving care, training and rehabilitating the patients (Organization).  And to create a learning environment in which new methods of coordinating and  integrating clinical and theoretical IPL are developed and tested  (Learning). | Three to six months after experiencing the SLW, students were positive about fulfilment of its goals.  Student colleagues have no problem saying to one another that they would like to accompany each other – and it is easy to make arrangements because all the students are together much of the day and therefore know what time various things happen. When  students from different professions work together with a patient, they are working on the same problem, but with different focuses – but they are not necessarily doing the same things. This forces the students to reflect when explaining to each other what they are doing and what they not are doing and why it is so. F = This clarifying of acting and reflecting helps the students in learning about their own profession and the others’ professions. | QL: Focus group interview of two students. from each of the four professions (randomly chosen among members of the first three teams, three to six months after their stay at the SLW). Discussion  was about IPL, learning environment, responsibility and  independence.  Analysis of Interviews was through Systematic Text Condensation (Malterud,2003 ) | A  SLW |
| Flemming Jakobsen, Kristian Larsen & Torben Baek Hansen (2010)  This is the closest I have come to being compared to a doctor: Views of medical students on clinical clerkship in an Interprofessional Training Unit  Medical Teacher; 32: e399–e406 | Denmark Holstebro & Aarhus  N= Med stud  55 filled in questionnaire;  22 interviewed.  ITU= interprofessional Training Unit  IPC was 2 to 9 days with nursing, PT and OT students. | Aims were: (1) to investigate to what extent the medical students’ stay in the ITU contributes to developing the student’s professional knowledge and capability  (2) and to what extent this stay contributes to teaching the student IPC (learning about other professions, working procedures in a ward, ability to work together & communicate with other professions) and  3) to evaluate the teaching and learning environment | Medical students in the ITU developed their *professional knowledge and capability* simultaneous with the learning of *interprofessional collaboration*.  Students valued the *teaching methods* because they were in the forefront and treated as professionals.  Students demanded more homogeneous instruction and a better introduction to the ITU.  F: med students felt equal in status with other students and the learning environment was safe.  F process: clinical tutors directed questions of stud to students who would be in that professional role in practice, the students were helped in their growth of professional identity. F process: teaching style of the clinical tutors with regard to providing time for reflection. F Process: authenticity of the context (working procedures regarding rounds, taking responsibility for treatment, communicating with patient) and practical tasks. | QT  Questionnaire regarding medical rounds, daily IP meeting, learning about own and other professions, overall assessment of learning in ITU  QL Asked for 3 statements (what was most important learning), then interviews  PBL patient centred | Thistlethwaite: KP 1  SLW |
| Zahra Keshtkaran, Farkhondeh Sharif, Masoume Rambod (2014)  Students' readiness for and perception of inter-professional learning: A cross-sectional study  Nurse Education Today 34; 991–998 | Iran, Shiraz  N= 250 undergraduate students | Correlational, cross-sectional study conducted on medical, nursing and science in surgical technology students to investigate their readiness for and perception of inter-professional learning and their relationship among healthcare students. | Medical students had the lowest scores on readiness for IPE and nursing students had the highest. Within readiness, the medical students had highest scores on understanding of roles and responsibilities and lowest on teamwork and collaboration. There were no gender-related differences. Readiness for IPE increased gradually from year 1 to year 4 and then decreased in all students. Med stud had higher mean scores on IEPS than nurs. Facilitators at individual level – Being a nursing student, year 4 of education. | RIPLS  IEPS | PBL for med stud, not nurs.  Gillan KP2a |
| Nana Kururi, Takatoshi Makino, Hiroko Kazama et al. (2014)  Repeated cross-sectional study of the longitudinal changes in attitudes toward interprofessional health care teams amongst undergraduate students  J Interprof Care; 28(4): 285–291 | Japan, Maebashi  N = 118 1^st^ year and 83 3^rd^ year students from dietetics, nursing, pharmacy and physiotherapy. | To investigate the implications of IPE comprising of a lecture style for first-year students and a training style for third-year students, the change pattern of attitudes toward health care teams was examined longitudinally in pre-qualified students. | The attitude to health care teams of students increased significantly after practice training in year 3 as compared to didactic training in year 1. The mean score did not change significantly from that at entry into the course. This means that the IPE training was not effective. | QT: modified ATHCTS pre and post first and third year. | SCL  Gillan KP2a |
| Jane Mikkelsen Kyrkjebø, Guttorm Brattebø & Hilde Smith-Strøm (2006)  Improving patient safety by using interprofessional  simulation training in health professional education  J Interprof Care, October; 20(5): 507 – 516 | Norway, Bergen  N= 12 students Med, Nurs and Intensive care Nurs | Pilot testing of a simulated training program in interprofessional student teams. Four teams each comprising of one medical, nursing and intensive nursing student, were exposed to two simulation scenarios twice. | F- process level =   - opportunity for reflection - training in team skills     B - individual level =   - focus on professional knowledge rather than team work - expectations of the other profession or role confusion | QL: FG were used to evaluate the program. | PBL, A  Thistlethwaite & Moran:  KP 2b |

| Heather K. Spence Laschinger and Wane Weston (1995)  Role Perceptions of Freshman and Senior Nursing and Medical Students and Attitudes Toward Collaborative Decision Making  J Professional Nursing, Vol 11, No 2 (March-April):119-128 | Canada, Ontario  N = students from  Med 1^st^ yr 64;  Med 4^th^ yr 34;  Nurs 1^st^ yr 50;  Nurs 4^th^ yr 59. | To identify gaps in perceptions of others' role. And the relationship between the degree of congruence in perceptions of each other's role and attitudes toward collaborative nurse physician patient care decision-making was also investigated. | Nursing students of both years were significantly more positive about physician-nurse collaboration than medical students. Gaps in perceptions of each other’s roles were significantly negatively related to attitudes toward collaborative patient care decision making. Facilitators for IPC at individual/cultural level – being a nurse. Barriers for IPC at cultural level - Gaps in perceptions of each other’s roles . | | QT | A |
| --- | --- | --- | --- | --- | --- | --- |
| Hanna Lachmann, Sari Ponzer, Unn-Britt Johansson, Lina Benson & Klas Karlgren (2013)  Capturing students' learning experiences and academic emotions at an interprofessional training ward  J Interprof Care, 27:2, 137-145, DOI:  10.3109/13561820.2012.724124 | Sweden, Stockholm  Med 11  Nurs 17  OT 5  PT4 | To investigate the learning experiences and academic emotions in authentic patient situations and which activities contributed to collaboration and creation of team knowledge (= trialogical activities). | Students were given mobile phones to answer questionnaires during the day, about their activities and academic emotions. Academic emotions are analysed using the four channel model (inspired by Csikszentmihalyi).  Students experienced flow (high challenge and high competence) when working close to the patient and in 54 % of these occasions in on-going collaboration with other students. | QL: questionnaires with free text answers | | A  SLW  Trialogical activities |
| Marie Lidskog, Anna Löfmark, & Gerd Ahlström (2008)  Learning about each other: Students’ conceptions before and after interprofessional education on a training ward  J Interprof Care, October; 22(5): 521–533 | Sweden  N= All female students except 1 male; 2^nd^ yr 4 SW  final yr Nurs 6  Final yr OT 6  3 weeks on a training ward. | To investigate similarities and differences in how student nurses, student OT and student social workers perceived their own and the other professions and in the students’ conceptions before and after clinical education on an IP training ward where the patients were older people. | This study shows that the perceptions of students with regards to other professions and their professional roles change for the better. There is also confirmation of some of their prejudices.  Only the two out-groups indicated that the nurse sometimes takes care of the patient too much, infringing the patient’s autonomy (category 6). | QL  Phenomeno-graphic analysis | | PBL patient centred, A  SLW |
| Marie Lidskog, Anna Löfmark & Gerd Ahlström (2009)  Learning through  participating on an interprofessional training ward  J Interprof Care, 23:5, 486-497, DOI: 10.1080/13561820902921878 | Sweden, Orebro  Nursing home with 8 units.  16 students included from  Nursing, OT (in their final yr) and  Social worker (SW, in their 2^nd^ yr). | Case study design to investigate learning on the ward within a socio-cultural context using concepts in the social theory of learning (communities of practice= CoP) -Wenger. | Students perceived to have learned to collaborate with other students/professions and thought it was valuable and enjoyable. The general activities on the ward were sometimes described as irrelevant for the learning, especially for SW. Three themes could be distinguished in CoP: ‘caring for the patients’ – sharing profession specific knowledge in relation to joint work; ‘being in a situation designed for learning’ - discussing each other’s’ professional perspective; ’profession specific training’- some understood CoP to be about performing profession specific tasks and they objected to the other focus . | QL interviews with students, and observations of work and reflection;  Focus groups with teachers regarding students learning on the ward. | | A: active student participation.  SLW |
| Per Lindblom, Max Scheja, Eva Torell, Per Åstrand & Li Felländer-Tsai (2007)  Learning orthopaedics: Assessing medical  students' experiences of interprofessional training  in an orthopaedic clinical education ward  J Interprof Care, 21:4, 413-423, DOI: 10.1080/13561820701401346 | Sweden, Stockholm  134 Med (75%) participated in assessment of their experiences of IP care which was performed with students from Nursing, OT and PT  Patient satisfaction was also investigated. | This study aimed to assess how medical students experienced IP care in an orthopaedic ward, at the same time investigating patient satisfaction. The hypothesis was that medical students could accomplish learning goals for orthopaedic and medicine in parallel with performing care that patients report to be satisfactory. | Before taking the SLW, medical students thought that it would be difficult to switch from being a medical student to providing general patient care. After the SLW the students did not corroborate this difficulty, though in some groups the general care had been experienced as more burdensome than in other groups. Students indicated that the SLW training had provided them with a good insight how a ward was run and their general learning goals had been fulfilled.  Patients rated the SLW as good to excellent. | QT: post SLW questionnaire to assess learning experiences from training in an IP team, the focus on medicine and orthopaedics and their previous experience working in Healthcare.  QT: patients were given a hospital evaluation form adapted to focus on communication with the medical students. | | A  SLW |
| Celia P. MacDonnell, Pharm D,S. V. Rege et al.(2012)  Instructional Design and Assessment. An Introductory Interprofessional Exercise for Healthcare Students    Am. Journal of Pharma. Education; 76 (8) Article 154. | N= 251:  83 Med stud 2^nd^ -year  67 Nurs stud 4^th^ -year  101 Pharm stud 3^rd^ -year | Evaluation of HC students’ perceptions of an introductory IP exercise and their team dynamics.  Teams alternated between working on patient cases focusing on a disease and on evaluation of standardized patients, being given only the patients’ health information. | Readiness for IPC increased after IPL intervention (1 day workshop) in terms of increased understanding of professional roles and positive attitude towards IPL.  The faculty members and standardized patients reported that the students worked as a cohesive unit and demonstrated good team communication. | A faculty member and standardized patient evaluated the students using a teamwork global rating scale. | | PBL, simulation patient |
| Takatoshi Makino, Hiromitsu Shinozaki, Kunihiko Hayashi, Bumsuk Lee, Hiroki Matsui, Nana Kururi et al. (2013)  Attitudes toward interprofessional healthcare teams: A comparison  between undergraduate students and alumni  J Interprof Care; 27: 261–268 | Japan, Maebashi  N=  501 (257 Nursing) undergraduate and 213 (101 Nursing) alumni | Cross-sectional descriptive study which employed  the modified attitudes toward health care teams scale (ATHCTS) to examine the relationship between exposure to clinical practice and the attitudes toward IP healthcare  teams. | Attitude towards health care teams is significantly poorer in students (nursing, physical therapy, occupational therapy, laboratory sciences) who have completed their graduation (around one year after graduation) in comparison with third year students after undertaking the same IPE training. Barrier at organizational/cultural level – Exposure to clinical practice. | QT:  Modified ATHCTS | | SCL  Gillan KP2a |
| Ruth McCaffrey, Rose Marie Hayes et al. (2012)  The effect of an educational programme on attitudes of nurses and medical residents towards the benefits of positive communication and  collaboration  Journal of Advanced Nursing 68(2), 293–301. | USA, Florida | Study conducted on medical residents and nurses. Undergoing an IPE initiative consisting of didactic teaching of communication skills followed by weekly meetings of the interprofessional teams. | Significantly improvement in attitude towards physician-nurse collaboration and communications among both residents and nurses. Facilitator of readiness for IPC at process level: IPE initiative with didactic session followed by regular IP team meetings. | QT: Jefferson Scale of Att toward Phys-Nurse Collaboration  and  Communication scale (Vazirani et al, 2005). | | Gillan KP2a |
| McFadyen, A. K.; Webster, V. S.; Maclaren, W. M.; O'Neill, M. A. (2010)  Interprofessional attitudes and perceptions: Results from a longitudinal controlled trial of pre-registration health and social care students in Scotland  J Interprof Care, 24(5), Sep, 549-564. | Scotland, Glasgow  N=  Control 260  Experimental 313 in year 1 start  To  Control 64  Experimental 120 at end year 4 | To assess the impact of a 4 year IPE intervention on the attitudes and perceptions of HC and social care stud with respect to IP ideals. | R= The RIPL is initially high, then a decline in the experimental group (EG) compared to the control group, followed by a relative improvement in scores in the EG.  F = teaching and learning strategies with problem oriented curriculum as positive for RIPL. | QT  RIPLS  IEPS  Control group is group before start intervention | | SCL  Gillan KP2a  Thistlethwaite: KP 2a |
| McGrail KA, Morse DS, Glessner T, Gardner K. (2009)  "What is found there": qualitative analysis of physician-nurse collaboration stories.  J Gen Intern Med. Feb;24(2):198-204. | USA, Rochester  25 Med residents,  32 staff Nurses,  5 Physicians,  5 Nurse faculty | To analyse and describe the collaboration experiences of nurses and physicians/medical residents, in an larger effort to improve the interactions between physicians and nurses. | Collaboration triggers were:  patient care crises (when next steps of care were not clear, life threatening or had a likely bad outcome ) and  affective crises (an emotion on the part of the professional: worry and/or vulnerability, e.g. feeling inadequate, overwhelmed). | QL, narratives  + phenomeno-logical approach to develop a framework for collaborative competence  IPC | | SCL? |
| Marion Mitchell, Michele Groves, Charles Mitchell,  Judy Batkin (2010)  Innovation in learning- An inter-professional approach to improving communication  Nurse Education in Practice; 10: 379-384 | Australia, Queensland  N=  13 3^rd^ yr Med  16 3^rd^ = last yr Nurs stud. | Pilot project to evaluate a tutorial format to promote IPE with nursing and medical students in mixed (nursing and medical students) versus  unmixed (nursing or medical students) tutorial groups. | Sense of belonging is less in an IP group than in a same profession group and this acts as a barrier for learning in IPE. Not knowing the students from the other profession meant that opening up did cost time.  Communication Style: more influenced by personal characteristics than by role or by (un-)mixed group.  An open communication style is a F, reserved style is a B, both on individual level. | QT: forced and open ended  Pre + post  QL: analysis of videotaped tutorials with qualitative ethnological technique. | | SCL |
| Morison, Sue; Jenkins, John (2007)  Sustained effects of interprofessional shared learning on student attitudes to communication and team working depend on shared learning opportunities on clinical placement as well as in the classroom  Medical Teacher, Vol 29(5), Jun, 464-470 | UK, Belfast  77 Med stud –no Shared Learning  78 Med stud – SL  35 Med+ 17 Nurs – SL + workplace | To explore the attitudes of students 1 year after their Shared Learning experience and to compare them with peers who had not participated in the SL programme. | Students who had undergone Shared Learning (SL) followed by a an IP clinical placement had better attitudes/readiness for IPC in comparison with only or no SL experience.  Medical students were reluctant to value learning that is not discipline-specific and particularly learning that is not examined. | QT with open ended questions  (no RIPLS but related to pilot programme)  IPL/IPE  communication, teamwork | | SCL |
| Virginie Muller-Juge, Stéphane Cullati, Katherine S. Blondon, Patricia Hudelson, Fabienne Maître,Nu V. Vu, Georges L. Savoldelli, Mathieu R. Nendaz (2013)  Interprofessional Collaboration on an Internal Medicine Ward: Role Perceptions and Expectations among Nurses and Residents  PLoS ONE 8(2): e57570. | Switzerland, Geneva  N= 14 residents and 14 nurses | This study investigated the perceptions of resident doctors and nurses regarding their professional roles in patient management. | Nurses’ expectations unmet by the residents are: explanation of actions/decisions, working in a team, listening to nurses, considering nurse’s opinion, recognizing nurses’ work, availability and knowing more about the nursing profession. Residents’ expectations unmet by the nurses are shared decision making, establishing a common goal for patient management, understanding the clinical situation, verifying prescriptions and medical decisions and exchanging information. There was a discrepancy between residents’ expectation of nurse autonomy in patient management. The nurses wanted more autonomy than the residents wanted to give them. | Semi structured interviews and scenario’s | | PBL  SLW |
| Douglas L. Myhre, Wayne Woloschuk and Jeanette Somlak Pedersen (2014)  Exposure and attitudes toward interprofessional teams: a three-year  prospective study of longitudinal integrated clerkship versus  rotation-based clerkship students  J Interprof Care; 28(3): 270–272 | Calgary, Canada  N = 213 medical students | Comparison of longitudinal-integrated versus rotation-based clerkship in exposure to and attitudes toward IP teams.  Assessing attitudes toward IP teams is important, because attitudes impact clinical performance. | Medical students who underwent Longitudinal Integrated Clerkships (thus having more exposure to interprofessional teams) had a significantly better attitude to IP teams in comparison with students in traditional rotation clerkships including rural, regional and urban (lower exposure to IP teams). | QT: survey pre and post clerkship | |  |
| Nadolski GJ, Bell MA, Brewer BB, Frankel RM, Cushing HE, Brokaw JJ. (2006)  Evaluating the quality of interaction between medical students and nurses in a large teaching hospital  BMC Med Educ. Apr 25;6:23. | USA  68 Med stud  (25.4% response, 61 white and 35 female)  99 Nurses (predominantly white + female) | Assess perceptions medical students hold of their interactions with nurses and other health care team (HCT) members during 3^rd^ yr clinical rotations. Nurses were surveyed about their interactions with med students and other HCT members in past 12 months. | In health care teams, medical students were perceived to interact with residents the best and with nurses the worst. Conversely, nurses interacted with other nurses the best and with medical students the worst.  The interpersonal sensitivity score of medical students was significantly greater than that of nurses, whereas the hostility score (B) of nurses was significantly greater than that of medical students. | QT, survey of dimensions:  4 communication  3 relationship  2 interpersonal sensitivity and hostility.  Readiness for IPC | | PBL |

| Nisbet, Gillian; Hendry, Graham D.; Rolls, Gary; Field, Michael J. (2008)  Interprofessional learning for pre-qualification health care students: An outcomes-based evaluation  J Interprof Care, 22(1), Jan,  57-68. | Australia, Sydney  N= 16:  Med stud 4^th^ yr,  Nurs stud in final yr. | Evaluate IPE programme’s goals:   - Explain roles of HC professional in team; - Show positive attit toward IPC Patient Care; - Effectively communicate + collaborate in IP Team | Students perceived increased understanding of professional roles, knowledge of teamwork  (relevance, dynamics, roles within, barriers) and increased competence in communication skills.  F= Recognition of barriers to effective HCT  B= feeling intimidated by some doctors  B= conflict between professions in how to manage patient care and not valuing others’ opinions.  Some lecturers and tutors neg influenced their perceptions of other professions; Timetable issues;  Although HCT members participated, stud see doctors as leader of clinical team. | QT: perceptions and satisfaction  QL: attitudes in interview  Multi method evaluation of IPL  Biggs: SOLO taxonomy | SCL  Thistlethwaite & Moran:  KP 2b |
| --- | --- | --- | --- | --- | --- |
| Birgitte Nørgaard, Eva Draborg, Erik Vestergaard, Eca Odgaard, Diddecramer Jensen & Jan Sørensen (2013)  Interprofessional clinical training improves self-efficacy of health care students  Medical Teacher; 35: e1235–e1242 | Denmark, Kolding  A quasi-experimental study with an intervention group (239 students)  and a control group (405 students). | 2 weeks in ITU: students learn from each other, develop inter- and uni-professional competence based on authentic patients’ health problems. IP reflection, both informal & formal (led by facilitator, student, or a vicar with specific ethical knowledge) | Measurement of perceptions of students about their ability to:   1. Collaborate with other professions in planning goals and actions for patients and for rehabilitation in an inpatient ward 2. Identify the functions of other professions in relation to inpatient care 3. Clearly assess and describe patients’ needs and problems, so that other professions can engage in a dialogue about goals and actions.   IPE is the intervention which has brought about a positive change in perceptions 2+3 and as such concerns Readiness for IPC. | QT (web based questionnaires, 4 questions – Parle, 1997) | PBL patient centered  SLW |
| Sujay Pathak, Christine G. Holzmueller, Karen B. Haller and Peter J. Pronovost (2010)  A Mile in Their Shoes: Interdisciplinary Education at the Johns Hopkins University School of Medicine  American Journal of Medical Quality 25(6) 462–467 | USA, Baltimore  N=  3 stud 4^th^ yr and  1 stud 3^rd^ yr. | A 2-week interdisciplinary elective course was developed after a needs assessment and piloted. Knowledge and competency survey to evaluate achievement of the course objectives. | IPE intervention had a positive effect on students’ perceptions of comfort with approaching non-physicians about patient care issues and understanding of the common challenges non-physicians face.  Having “preceptors” in IPE interventions is a F for IPE. | QT pre-course and post-course | PBL |
| Katherine C. Pollard & Margaret E. Miers (2008)  From students to professionals: Results of a longitudinal study of attitudes to pre-qualifying collaborative learning and working in health and social care in the United Kingdom  J Interprof Care, August; 22(4): 399–416 | UK  N = 414 students:  275 students educated on the IP curriculum,  139 graduated on previous uni-professional curricula. | To compare IP and non IP groups:   - Communication, Teamwork; - Attitudes towards IPE; - Perceptions of quality of IP interaction between other health and/or social care professionals; - Perceptions of the quality of (IP) relationships with colleagues from own and other professions. | Prequalifying IPE has a positive effect on individuals’ attitudes towards and perceptions of, IP working. This effect appears to be maintained, and even strengthened, once they are practicing as qualified professionals.  More mature age and prior HE qualifications led to weaker correlations between the scales. | UWE IPQ, consisting of 4 scales | PBL setting  Gillan KP2a |
| Sari Ponzer, Uffe Hylin, Ann Kusoffsky, Monica Lauffs, Kirsti Lonka, Anne-Cathrine Mattiasson & Gun Nordström (2004)  Interprofessional training in the context of clinical  practice: goals and students’ perceptions on clinical  education wards  Medical Education; 2004; 38: 727–736 | Sweden, Stockholm  Med 210  Nurs 470  OT 98  PT 184 | This study investigated how students perceived their professional and Interprofessional training. | Most students (64%, n= 617), perceived their professional role more clearly after the SLW ; however, 29% did not differ after SLW from before in a retrospective account (checked for bias).  Students from all professions reported a significant increase in knowledge of others’ professional role during the SLW. Also their insight in the importance of communication for teamwork, their awareness of the patient’s role and of ethical aspects of healthcare had increased significantly. OT students rated both items significantly higher than students from other professions. | QT, post - asking for perceptions of own professional role clarity, others’ professional role, the crucial communication for good team work, patient participation and attitudes towards IPE in SLW and ethical aspects of healthcare. | A: students should apply problem solving skills and make use of the knowledge and skills from other students.  SLW |
| Scott Reeves, Della Freeth, Peter McCrorie & David Perry (2002)  ‘It teaches you what to expect in future’: interprofessional learning on a training ward for medical, nursing, occupational therapy and physiotherapy students  Medical Education;36:337–344 | UK, London  N= 36;  6 teams of 6 stud  (2 Nurs, 2 Med,  1 OT, 1PT) | Multi-method evaluation of an interprofessional training ward:   - experiences of IPC of staff, students and institutions involved in this initiative. - considers the impact of this collaborative work on patient care. - longer-term effects of this form of IPE from 1-year follow-up questionnaires completed by former training ward students   Assessment criteria: ability to assess their own IPC; background knowledge of patients; and ability to plan care interventions and treatment strategies. | F = authenticity of context, team reflection sessions.  B = Lack of participation in team duties by the medical students, combination of profession-specific and interprofessional learning objectives to be achieved in the same placement create confusion and tension about whether the focus should be on the former or the latter.  B culture =   - Stud. from all professions labelled team duties as ‘nursing work’. - Roles of other professions were not clarified / defined enough. - Placement facilitation required a great amount of time from facilitators. - Tutors had little time to be role models, they worked parallel, mostly exchanging patient information.   Improvement suggested by former students, all F= process:   - longer placements; - pre-training ward preparation to align expectations about student roles on the ward; - more consistent facilitation to overcome difficulties with team functioning; - style of facilitation: providing direction and working with students encourages accountability for team work. | QT  Questionnaires for training ward (n=34) and non- training ward patients (n= 34);  Students: perceptions of IPE pre+ post + 1 yr after.  QL, action research,  - group interview with each team  - Individual interview with 10 facilitators | PBL patient centred  Thistlethwaite & Moran:  KP 2b  SLW |
| Rosenfield D, Oandasan I, Reeves S. (2011)  Perceptions versus reality: a qualitative study of students' expectations and experiences of interprofessional education  Medical Education, ,May;  45(5):471-7. | Canada, Toronto    N 2007 = 23 stud who finished 1^st^ year,  4 Med; 6 OT  8 Dental,3 Pharm  2 Social work  N 2008= 12 Med finished 1^st^ yr. | To evaluate and compare student experience of IPE event in 1^st^ year and perceptions and experiences in the 2^nd^ year. | After the big event, students advised for effective IPE:  F=To employ small group sessions  F=With active learning (not lectures);  F= In a longitudinal setting;  F= Well integrated in the curricula. | QL: Focus groups  IPL evaluation | SCL  Thistlethwaite: KP 1 |
| Rudland, Joy R.; Mires, Gary (2005)  Characteristics of doctors and nurses as perceived by students entering medical school: implications for shared teaching  Medical Education, Vol 39(5), May, 448-455. | New Zealand, Dundee  N= 601 Med stud 1^st^ yr | Examination of students’ perceived professional characteristics and background of doctors and nurses upon entering medical school.  These perceived impressions, which may reflect societal  misconceptions regarding the roles and responsibilities  of nurses within a modern health care system, may have  an impact on the success of early IPE initiatives in undergraduate curricula. | Characteristics: caring, confident, dedicated, detached, do-gooder, good communicator, indecisive, arrogant.  B= Medical students perceive nurses to be more caring than doctors but to have less positive status in society and to be less competent and academically weaker than doctors(B – Stereotype, cultural).  Medical students are generally positive about shared learning (SL) at an early stage.  Principal advantages of SL cited were increase in understanding of the roles of all professions, improvement in team working and better care for patients.  Disadvantages of SL most cited were reduced relevance for each profession, reduced pace of learning, reinforcement of negative stereotypes and confusion of roles. This means that SL could be both a F and a B. | QT : questionnaire developed by Carpenter (1995) re. characteristics professions; background, own professional identity  QL: which are the sources for difficulties between D and N and how can they be overcome?  N= 389 | SCL |
| Irma Ruebling, David Pole, Anthony Paul Breitbach, Alfred Frager et al. (2014)  A comparison of student attitudes and perceptions before and after an  introductory interprofessional education experience  J Interprof Care; 28(1): 23–27 | USA, St Louis  N= 305 stud and 202 Control group | To determine attitudes and perceptions of students toward collaborative learning in an interprofessional context. Students completed a questionnaire regarding attitudes and perceptions toward interprofessional collaboration before and after  an introductory IPE course. | F = positive attitude for Shared learning became more positive after the course with perceived benefits such as facilitating subsequent working relationships, improving teamwork, and ultimately improving patient care. | QT (pre and post):   - RIPLS - UWEIQ (9 items) | SCL  Gillan KP2a |
| Dimitrios Siassakos, Christina Timmons, Florence Hogg, Mathias Epee, Lisa Marshall & Timothy Draycott (2009)  Evaluation of a strategy to improve undergraduate  experience in obstetrics and gynaecology  Medical Education:43: 669–673 | UK, Bristol  N = 27 Med stud | Study aimed to assess whether student reactions translated into both better IP attitudes and potential for improved recruitment.  Participation in IP research teams and simulation exercises. New teaching sessions were delivered by midwifery lecturers and midwifery students as tutors, to improve the learning experience of medical students. | This study investigates simulation-based obstetric emergency training as an IPL intervention. There was an improvement in the perceptions of interprofessional teamwork and communication and  an improvement in IP relationships. | QT:  UWE IP questionnaire comprises 4 validated attitude scales. | SCL  Gillan KP2a |
| Solomon P.(2011)  Student perspectives on patient educators as facilitators of Interprofessional education  Medical Teacher;33(10):851-3 | Canada, Ontario  FG: N= 27  QL: N= 138  Med: 1^st^ yr, other students senior level. | Students perceptions of patient facilitated IPE | F= Patient-facilitated IPE event has positive effect on readiness for IPE. Having “patient educators”.  Students see IPE as a positive learning experience, to advocate their specific role, to learn from other students.  F= giving back to the patients who gave their personal stories and experiences in health care. (Table 1, p 252) | QL  Critical incident questions (open end) to capture impressions following IPE. | PBL |
| Paul Stepney, Ingrid Callwood,  Flora Ning and Kevin Downing (2011)  Learning to collaborate: a study of nursing students’ experience of  inter-professional education at one UK university  Educational Studies Vol. 37,  No. 4, October, 419–434 | UK Wolverhampton,  *N* = 105; sample = 29 students’ experience of the module in two stages.  Nursing and social work students.  Readiness for IPC. | (1) To establish students’ knowledge, understanding and attitudes to collaborative  working, both at the beginning and end of the module.  (2) To explore the ways in which the module impacted upon students’ professional  approach to collaborative working. | B = after the module, most students perceived professional tribalism as a problem of collaborative working.  B = postgraduate students highlighted problems associated with status/power differentials,  B = fear about loss of professional identity (71.4%)  B = feeling threatened (85.7%), insufficient time or trust was highlighted by 62.5% of undergraduate students as a problem of collaborative working.  F = Stud acknowledged that achieving consensus is integral to collaborative working. Sixteen of the students felt that their confidence was developed whilst on the module. F = Alongside confidence, collegiality was identified as being an important element of collaborative working and then stud can relax more. F = Giving people time to socialise, so that they can learn about each other in order to develop collegial relationships is also identified as important.  F process = "having teachers/lecturers from your own and other professions" | QT - Stage 1:  to examine pre and post module differences and between the two  seminar groups.  QL - Stage 2: semi-structured interviews at  end of the module with the same sample of students. | SCL |
| Nancy M. Tofil, Jason L. Morris, and others (2014)  Interprofessional Simulation Training Improves Knowledge  and Teamwork in Nursing and Medical Students During  Internal Medicine Clerkship    Journal of Hospital Medicine Vol 9 | No 3 | March 189- 192 | USA, Alabama  N=  72 Med stud 3^rd^ yr; 28 senior Nurs stud.  10 months, 4x1 hr, 4 cases. | Identical pre- and post-tests were given to medical  and nursing students.  Case-specific knowledge was assessed with multiple choice items.  Self-efficacy related to professional roles and attitudes toward team communication were each assessed. | Simulation-based IPE increased self-efficacy and understanding of other's professional role. That means the readiness for IPC increased because of the IPE intervention.    Both groups of students had the greatest improvement in “confidence to correct another healthcare provider at bedside in a collaborative manner.” | QT (regarding cases) + open ended evaluation quest about simulation effectiveness + areas for improvement.  Pre and post comparison | PBL |
| Tunstall-Pedoe S, Rink, E, Hilton S. (2003)  Student attitudes to undergraduate interprofessional education  J Interprof Care. May;17(2):161-172 | UK, London  N students=  176+176 Med  36+35 Physio  40+35 Radiol  8 Nurs | Common Foundation Programme (CFP) involves medical students learning alongside allied health and nursing students. | Students enter their Health Care Education with stereotyped views of each other (Barrier), and these views appeared to become more exaggerated during a Common Foundation Programme.  B process = Students felt that the CFP would enhance interprofessional working, but there were concerns that it forced them to learn irrelevant skills.  B= Students whose parents worked in health care, held stronger stereotyped views. (Lower readiness) | QT based on Carpenter (1995) | SCL, A |

| Mary van Soeren, Sandra Devlin-Cop, Kathleen MacMillan, Lindsay Baker, Eileen Egan-Lee, and  Scott Reeves (2011)  Simulated interprofessional education: An analysis of teaching and  learning processes  J Interprof Care; 25(6): 434-440 | Canada, Toronto  N = 152 clinicians (Nurs, SW, Med, OT, PT, Pharm)  +  101 Stud (Pharm, PT/OT, Nursing). | This study investigated the response of clinicians and students of different professions to simulated IPC situations as an IPL intervention. | Facilitators for IPL :   - Enthusiasm and motivation of participants - Acting out professional roles (own or others) - Realistic clinical scenarios - Facilitator style: not “imparting knowledge (teachers)” - Facilitators from different professions bring complementary perspectives and work best. | QL: observation (+Video taping ) of simulation and debrief.  FG with clinicians separate from students. | SCL  Thistlethwaite: Process evaluation. |
| --- | --- | --- | --- | --- | --- |

**Legend:**

Med= medical; Nurs= nursing; OT= Occupational Therapy; PT= Physical Therapy; stud= students;

QL = qualitative research; QT = quantitative research; IP = Interprofessional;

B= barrier to IPE; F= facilitator of IPE; RIPLS= Readiness for Interprofessional Learning Scale (Parsell & Bligh, 1999); IEPS= Interdisciplinary Education Perception Scale (Luecht et al, 1990); ATHCTS = Attitude Toward Health Care Teams Scale (Heinemann et al, 1999);

SLW = Student Led Ward; SCL = student centred learning; PBL = problem based learning; SG = Small group; A= pedagogical approach described in a paragraph.

In compliance with the reviews by Gillan et al. (2011), by Thistlethwaite et al. (2014) and by Thistlethwaite and Moran (2010) we classified the Kirkpatrick level of the studies in this review by the instrument they applied to evaluate the learning outcomes. In 18 studies instruments were used classified by Gillan at Kirkpatrick level 2a, namely the Readiness for Interprofessional Learning Scale (in 8 studies), the Jefferson Scale of Attitudes toward Physician-Nurse Collaboration (in 2 studies), the Interdisciplinary Education Perception Scale (in 3 studies), the Attitude Toward Health Care Teams Scale (in 4 studies), the University of West England Interprofessional Questionnaire (in 2 studies) or a combination of these instruments. Five studies in this review were classified by Thistlethwaite as Kirkpatrick level 2a. Three studies were categorized by Thistlethwaite and Moran as Kirkpatrick Level 2b. Regarding three studies (Bradley et al.; Carpenter; Cooper et al.) the classification of Thistlethwaite & Moran and of Gillan differs, as can be seen in the last column in this Appendix.
